# Insights into POT1 structural dynamics revealed by cryo-EM

**DOI:** 10.1371/journal.pone.0264073

**Published:** 2022-02-17

**Authors:** Emmanuel W. Smith, Simon Lattmann, Zhehui Barry Liu, Bilal Ahsan, Daniela Rhodes

**Affiliations:** 1 NTU Institute of Structural Biology, Nanyang Technological University, Singapore, Singapore; 2 School of Biological Sciences, Nanyang Technological University, Singapore, Singapore; Tulane University Health Sciences Center, UNITED STATES

## Abstract

Telomeres are protein-DNA complexes that protect the ends of linear eukaryotic chromosomes. Mammalian telomeric DNA consists of 5′-(TTAGGG)n-3′ double-stranded repeats, followed by up to several hundred bases of a 3′ single-stranded G-rich overhang. The G-rich overhang is bound by the shelterin component POT1 which interacts with TPP1, the component involved in telomerase recruitment. A previously published crystal structure of the POT1 N-terminal half bound to the high affinity telomeric ligand 5′-TTAGGGTTAG-3′ showed that the first six nucleotides, TTAGGG, are bound by the OB1 fold, while the adjacent OB2 binds the last four, TTAG. Here, we report two cryo-EM structures of full-length POT1 bound by the POT1-binding domain of TPP1. The structures differ in the relative orientation of the POT1 OB1 and OB2, suggesting that these two DNA-binding OB folds take up alternative conformations. Supporting DNA binding studies using telomeric ligands in which the OB1 and OB2 binding sites were spaced apart, show that POT1 binds with similar affinities to spaced or contiguous binding sites, suggesting plasticity in DNA binding and a role for the alternative conformations observed. A likely explanation is that the structural flexibility of POT1 enhances binding to the tandemly arranged telomeric repeats and hence increases telomere protection.

## Introduction

Telomeres are protein-DNA complexes that protect chromosomes from degradation by preventing end-to-end fusion and countering the end replication problem [1–3]. Mammalian telomeric DNA consists of 5′-(TTAGGG)n-3′ double-stranded repeats, followed by up to several hundred bases of single-stranded telomeric repeats forming a 3′ G-rich overhang (G-overhang) [1–4]. Telomeres shorten with every cell division, and while this progressive shortening is generally considered a hallmark of aging, in stem and cancer cells it can be countered by a dynamic interplay between the telomerase enzyme that synthesizes telomeric repeats and the telomere-binding protein complexes shelterin and CST (CTC1, STN1, TEN1) [5–8]. Consequently, telomeres and the protein complexes that regulate their structure or stability are intensively studied in attempts to develop therapies for cancer and age-related disease [4, 9, 10].

**Fig 1:**
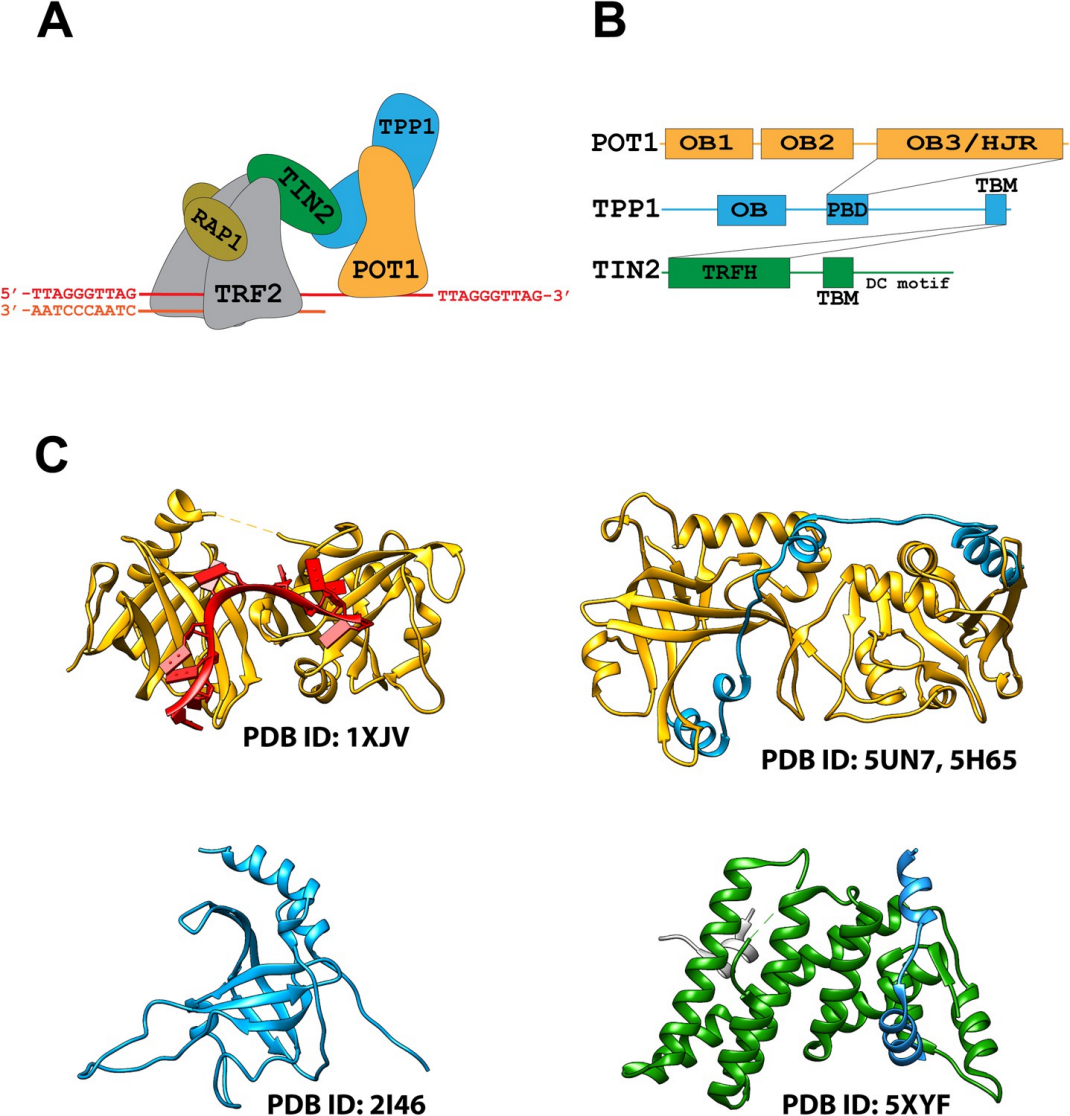
Composition and architecture of the shelterin complex. (**A**) Cartoon representation of the full shelterin complex POT1-TPP1-TIN2-TRF2(2×)-RAP1(2×) bound to a telomeric double-stand/singe-stand junction. (**B**) Interaction map for the shelterin subcomplex POT1-TPP1-TIN2. The OB3/HJR domain of POT1 (orange) interacts with the PDB of TPP1 (blue), while the TBM of TPP1 (blue) interacts with TIN2 through the TRFH domain of TIN2 (green). TIN2 also has a TBM and a DC motif. (**C**) Published crystal structures of domains of the POT1-TPP1-TIN2 shelterin subcomplex: POT1 (OB1 & OB2) (yellow) bound to the minimal telomeric sequence TTAGGGTTAG (red) (PDB ID: 1XJV) (top left), POT1 (OB3 & HJRD) (yellow) bound by the PBD of TPP1 (E266-L326) (blue) (PDB ID: 5UN7, 5H65) (top right), TPP1 OB domain (blue) (PDB ID: 2I46) (bottom left), and TIN2(green)-TPP1(blue)-TRF2(grey) interface complex (PDB ID: 5XYF) (bottom right).

Shelterin is a multiprotein complex that binds mammalian telomeres forming a protective capping structure [11–13]. It consists of the protection of telomeres protein 1 (POT1), the POT1 interacting factor TPP1, the telomeric repeat-binding factors 1 and 2 (TRF1 and TRF2) as well as RAP1 and TIN2 (Fig 1A) [11, 13, 14]. POT1 on the one end of the complex binds to the telomeric single-strand G-overhang, while homodimers TRF1 or TRF2 at the other end of the complex bind to the telomeric double-stranded repeats, and together, these proteins anchor the full shelterin complex onto the single-strand/double-strand junction (Fig 1A) [11, 15]. Additionally, POT1 complexed to TPP1 binds independently and sequence-specifically to multiple sites, coating the telomeric G-overhang [16, 17]. Finally, shelterin or sub-complexes containing at a minimum only the POT1 and TPP1 subunits can recruit telomerase through the TEL patch domain on TPP1, and hence regulate telomere elongation and prevent severe telomere instability [18–21].

The POT1 subunit consists of three oligonucleotide/oligosaccharide binding (OB)-folds and a Holiday Junction Resolvase (HJR) domain (Fig 1B) [22–25]. The ssDNA-binding domain (DBD) of POT1 is located at its N-terminus and consists of two adjacent OB folds (OB1 and OB2) (Fig 1B and 1C) [25]. The C-terminal half of POT1 contains the third OB fold (OB3) intertwined with the HJR domain and mediates the interaction to TPP1 through binding to the POT1-binding domain (PBD) of TPP1 (E266-L326) (Fig 1B and 1C) [23, 24]. TPP1 in turn interacts with TIN2 that forms a bridge to TRF1 and TRF2 (Fig 1) [26].

The telomeric decamer sequence 5′-TTAGGGTTAG-3′ is the minimal tight binding sequence for POT1 with a reported apparent K_d_ of 10 nM [25]. A crystal structure (PDB ID: 1XJV) of the N-terminal half of POT1 bound by this decamer sequence revealed that the two OB folds (OB1 and OB2) pack together forming a cavity along their axis where the single-stranded DNA ligand binds (Fig 1C) [25]. The interactions with the DNA strand are orchestrated mainly through the stacking of bases with hydrophobic residues, while the phosphate backbone is solvent exposed [25]. The structure also revealed that OB1 binds to a half-site consisting of the first six nucleotides (TTAGGG), while the adjacent OB2 binds to a half-site consisting of the last four nucleotides (TTAG) of the binding-site [25]. It was also reported that dT2, dA3, dG4, and dG5 of the ssDNA ligand contribute the most to binding, demonstrating that most of the DNA-binding affinity stems from the OB1 fold [25].

Currently, information on the three-dimensional structure of the shelterin complex, or any of the isolated full-length protein subunits is sorely lacking. Structural information is limited to crystal structures of some of the sub-domains of the shelterin subunits, or complexes of protein-protein interfaces (Fig 1C) [23–32]. Here, we report on our efforts to determine the structure of the shelterin sub-complex POT1-TPP1-TIN2(1–354) by cryo-EM. Our attempts to obtain the structure of the full shelterin complex (POT1-TPP1-TIN2(1–354)-TRF2-RAP1) were unsuccessful, likely due to high conformational heterogeneity. We have reconstructed two 3D maps: One at 7.9 Å resolution into which we can fit the full-length POT1 protein complexed to the PBD of TPP1. While we could unambiguously fit the structures of the POT1 OB1 and the POT1 OB3/HJR domain bound to the PBD of TPP1 into the cryo-EM map, densities for the remaining TPP1 and TIN2 proteins are missing. The second map at 9.6 Å resolution displays an alternative conformation for POT1, in which OB1 and OB2 occupy densities that are spaced further apart from each other, suggesting POT1 has a dynamic structure allowing the two OB folds to take up an alternative conformation. Finally, to test the hypothesis that an extended conformation of the two OB folds could affect DNA recognition and that binding was not exclusively restricted to the minimal continuous binding sequence TTAGGGTTAG, we performed DNA binding studies using telomeric ssDNA ligands in which spacers were introduced between the half-sites for OB1 and OB2. We find that ligands with spaced half-sites indeed bind with no significant loss of affinity. We speculate that the alternative orientations we observe for the POT1 OB folds might have important implications for the recognition of telomeric tandem repeats and telomere protection.

## Materials and methods

### DNA sequences and construction of dsDNA telomeric ligands

All DNA oligonucleotides (S1B Fig) were purchased from Integrated DNA Technology. For EMSA and WEMSA experiments, the anti-sense oligonucleotide ctelo was 5′-labelled with [γ-^32^P]ATP (6000 Ci/mmol, PerkinElmer) and T4 polynucleotide kinase (New England BioLabs) according to the manufacturer’s instructions. To generate dsDNA telomeric ligands, sense and anti-sense DNA strands at 2 μM concentration each were combined in annealing buffer (70 mM Tris-HCl (pH 7.6), 10 mM MgCl_2_). Reactions were heated at 100°C for 5 min on a PCR thermocycler and subsequently cooled to 25°C at a constant rate of -1.0°C/min.

### Expression of shelterin sub-complexes in insect cells using MultiBac

Full-length human POT1 (1–634) fused to a TEV protease cleavage site and an N-terminal His-10 tag was cloned into the pFL acceptor plasmid through Gibson assembly [33] along with an N-terminal Strep-tag II tagged variant of TPP1 (full-length (1–544), N-terminal truncation variant (87–544), or both N-terminal and C-terminal truncation variant (87–334)) also fused to a TEV protease cleavage site. Similarly, either full-length (1–451) or a C-terminal truncation variant (1–354) of TIN2 was cloned into the donor pUCDM plasmid. Cre-LoxP fusion reaction was used to recombine the acceptor and donor plasmids and the correct constructs were selected in the presence of ampicillin and chloramphenicol. The chosen multicistronic constructs were subsequently subcloned into the MultiBac bacmid and further selected by blue-white screening. Sf9 insect cells were transfected with the bacmid DNA and used to amplify the virus. The viral titer was determined by the end-point dilution assay. For expression of POT1-TPP1-TIN2 constructs, 700 ml Sf9 cell cultures (at 3×10^6^ cells/ml) were infected (MOI = 1.5) and cultured for 3 days in suspension at 27° C. Cells were harvested by centrifugation and pellets stored at -80°C for future use.

For expression of the TRF2 and RAP1 shelterin components, untagged full-length RAP1 (1–399) and full-length TRF2 (1–542) fused to an N-terminal Twin-Strep-tag and a TEV protease cleavage site were cloned into the pFL plasmid through Gibson assembly. The bicistronic construct was then inserted into the MultiBac bacmid and the remaining steps were implemented similarly as for the POT1-TPP1-TIN2 constructs.

### Purification of recombinant shelterin protein sub-complexes

A pellet of Sf9 cells expressing the POT1-TPP1-TIN2(1–354) construct was resuspended in lysis buffer (300 mM NaCl, 20 mM Hepes pH 7.5, 25 mM Imidazole, 5 mM MgCl_2_, 1 mM TCEP, DNase, 1× cOmplete™- EDTA-free Protease Inhibitor Cocktail, 0.1% (v/v) Triton X-100, 1 mM PMSF) and sonicated (40 amplitude, 3 s on/5 s off, 10 min), before being centrifuged at 25,000g for 1 hour. The clarified cell lysate was filtered and loaded onto a 1-ml HisTrap HP column at 1 ml/min flow rate. A salt wash was then performed with buffer containing 1 M NaCl, 20 mM Hepes pH 7.5, 25 mM imidazole, and 1 mM TCEP, followed by a wash with buffer containing 300 mM NaCl, 20 mM Hepes pH 7.5, 74 mM imidazole, and 1 mM TCEP. Elution was performed over a 10-column volume gradient to 100% Elution Buffer (300 mM NaCl, 20 mM Hepes pH 7.5, 750 mM imidazole, 1 mM TCEP) at 1 ml/min flow rate. The eluted sample was then supplemented with 0.5 mg TEV protease and dialyzed overnight in dialysis buffer (300 mM NaCl, 20 mM Hepes pH 7.5, 25 mM imidazole, 5 mM MgCl_2_, 1 mM TCEP) to reduce the imidazole concentration. The dialyzed sample was reloaded onto a HisTrap HP 1-ml column to remove the uncleaved protein, the TEV protease, and contaminants. The flowthrough was then concentrated to 100 μl and injected onto a Superdex 200 Increase 10/300 GL column pre-equilibrated with the Size Exclusion Buffer (300 mM NaCl, 20 mM Hepes pH 7.5, 10% (v/v) glycerol, 1 mM TCEP). The complex eluted around 13.5 ml and the purity as assessed by SDS-PAGE was found to be >95% (S2A Fig). The fractions corresponding to the peak were pooled and the complex dispensed into 100-μl aliquots (0.7 mg/ml), flash frozen in liquid nitrogen and stored at -80°C for future use.

For TRF2-RAP1, an Sf9 cell pellet was resuspended in lysis buffer (300 mM NaCl, 20 mM Hepes pH 7.5, 2 mM MgCl_2_, 0.5 mM TCEP, DNase, 1 mM Benzamidine, 0.1% (v/v) Triton X-100 and 1 mM PMSF) and sonicated (40 amplitude, 3 s on/5 s off, 10 min), before being centrifuged at 25,000g for 1 hour. The clarified cell lysate was filtered and loaded onto a 1-ml StrepTrap HP column at 1 ml/min flow rate. The immobilized protein was then washed (300 mM NaCl, 20 mM Hepes pH 7.5, and 0.5 mM TCEP) followed by a high-salt wash (1 M NaCl, 20 mM Hepes pH 7.5, and 0.5 mM TCEP) and eluted with elution buffer (300 mM NaCl, 20 mM Hepes pH 7.5, 0.5 mM TCEP, and 5 mM D-Desthiobiotin). The eluted sample was incubated overnight in the presence of 0.5 mg TEV protease and was then concentrated to 100 μl and injected onto a Superdex 200 Increase 10/300 GL column pre-equilibrated with the Size Exclusion Buffer (300 mM NaCl, 20 mM Hepes pH 7.5, 10% (v/v) Glycerol, 0.5 mM TCEP). The TRF2-RAP1 complex eluted around 11.3 ml and the purity as assessed by SDS-PAGE was found to be >95% (S2A Fig). The fractions corresponding to the peak were pooled and the sample was dispensed into 50-μl aliquots (1.25 mg/ml), flash frozen in liquid nitrogen and stored at -80°C for future use.

### Reconstitution of fully assembled shelterin complex

The fully assembled shelterin complex (POT1, TPP1, TIN2, TRF2 and RAP1) was produced by independently overexpressing POT1-TPP1-TIN2 and TRF2-RAP1 as two separate sub-complexes and purifying each sub-complex to high purity (S2A Fig). For the POT1-TPP1-TIN2 shelterin sub-complex, a construct containing full-length POT1, full-length TPP1 and a C-terminally truncated allele of TIN2(1–354) was chosen as it yielded the highest expression of the complex, and because the C-terminus of TIN2 contains no known protein-interacting domains [34]. The full shelterin complex was then assembled by combining the two purified sub-complexes, followed by fractionation by size exclusion chromatography.

### Analytical size exclusion chromatography of shelterin complexes

Analytical size exclusion chromatography was used to assess the binding of telomeric ssDNA ligands to the POT1-TPP1-TIN2(1–354) complex. It was performed using a Superose 6 Increase 10/300 GL column pre-equilibrated with a 300 mM NaCl, 20 mM Hepes pH 7.5, and 1 mM TCEP buffer. 600 pmol of purified recombinant POT1-TPP1-TIN2(1–354) was injected onto the SEC column alone, or after incubation (30 min, 4° C) with 1200 pmol of the telomeric ssDNA ligand GGTTAGGGTTAG (sstelo64). For comparison, 600 pmol and 1200 pmol of the telomeric ssDNA ligand were injected separately, and the elution profiles were superimposed and analyzed (S3 Fig). To test the capacity of the TIN2(1–354) variant to bridge TRF2/RAP1 and reconstitute the full shelterin complex (POT1-TPP1-TIN2(1–354)-TRF2(2×)-RAP1(2×)), 200 pmol POT1-TPP1-TIN2(1–354) or 400 pmol of TRF2-RAP1 were first injected alone onto a Superdex 200 Increase 10/300 GL column pre-equilibrated with a 300 mM NaCl, 20 mM Hepes pH 7.5, and 1 mM TCEP buffer. Subsequently, 200 pmol POT1-TPP1-TIN2(1–354) was combined with 400 pmol of TRF2-RAP1 and equilibrated (30 min, 4° C) prior to being injected onto the SEC column. The chromatograms from the three separate runs were then superimposed and analyzed for complex formation (S2B Fig).

### Direct telomerase assay

The human telomerase was overexpressed and purified as previously described [35]. Purified recombinant shelterin protein complexes (POT1-TPP1-TIN2(1–354), TRF2-RAP1 or POT1-TPP1-TIN2(1–354)-TRF2-RAP1) at concentrations from 100 to 5 nM (20^1/7^-fold serial dilution) were incubated with 10 nM telo666 telomeric ligand (S1B Fig) in a total volume of 10 μl telomerase assay buffer [50 mM Tris-HCl (pH 8.0), 50 mM KCl, 1 mM MgCl_2_, 4 mM 2-mercaptoethanol, 500 μM each of dATP and dTTP, 20 μM dGTP and 20 nM [α-^32^P]dGTP (6000 Ci/mmol, PerkinElmer)]. The binding reactions were pre-equilibrated at 30°C for 30 min. Telomeric DNA extension reactions were initiated by the addition of purified recombinant telomerase and incubated at 30° C for an extra 30 min. Reactions were terminated by addition of an equal volume of deionised formamide. After addition of proteinase K (0.5 mg/ml), the reaction mixtures were further incubated at 37° C for 20 min. The reaction products were heat-denatured for 10 min at 95°C and one-half of the reaction volume was resolved (17 W, 65 min) on a pre-electrophoresed (17 W, 60 min) denaturing (7 M urea, 0.5× TBE) 8% polyacrylamide (19:1 mono:bis ratio) gel. Gels were subsequently dried onto a positively charged Hybond N+ nylon membrane (GE Healthcare) and phosphorimaged (Typhoon FLA 7000).

### Activity analyses of shelterin complexes

To establish that the purified POT1-TPP1-TIN2(1–354) sub-complex produced by over expression was fully functional, we analyzed its ability to assemble into a fuller shelterin complex (S2B Fig), its DNA binding properties (S3, S6 and S7 Figs), as well as its effect on the enzymatic activity of the telomerase enzyme (S2C Fig).

Analysis of the elution profile for the telomeric ssDNA ligand (sstelo64) alone or combined suggests the complex is binding (S3 Fig) and was also verified by (W)EMSA (S6 and S7 Figs). Additionally, TIN2(1–354) appears to effectively bridge the POT1-TPP1 and TRF2-RAP1 components forming a larger complex as indicated by the position of the elution profile when combining the POT1-TPP1-TIN2(1–354) and the TRF2-RAP1 complexes (S2B Fig). Lastly, titrating the telomerase ligand (telo666, S1B Fig) with increasing concentrations of the POT1-TPP1-TIN2(1–354) shows a marked dose-dependent augmentation in telomerase processivity (S2C Fig), consistent with previous observations [27]. Similarly, a titration with the full shelterin complex also manifested an increase in telomerase processivity, although with a greater stimulatory effect at low concentrations in comparison to the POT1-TPP1-TIN2(1–354) complex alone. As a control, the TRF2-RAP1 complex alone had no effect on telomerase processivity. In summary, the purified full shelterin complex as well as the POT1-TPP1-TIN2(1–354) shelterin sub-complex we have produced appear to be fully functional in shelterin assembly and DNA binding, and are proficient in telomerase recruitment to the G-overhang for telomere repeat synthesis. Therefore, both complexes were judged suitable for cryo-EM structural studies.

### Grid preparation and data collection parameters

Frozen 0.7 mg/ml aliquots of POT1-TPP1-TIN2(1–354) were thawed and dialyzed overnight at 4°C in a dialysis cap against a glycerol-free buffer (200 mM NaCl, 20 mM Hepes pH 7.8, 1 mM 2-mercaptoethanol). The sample was further diluted to 0.05 mg/ml, and 3 μl drops were applied onto 1.2/1.3 UltrAuFoil Grids inside a Vitrobot Mark IV (Thermo Fisher Scientific) chamber maintained at 100% humidity and 4°C. The grids were blotted for 2 seconds at 0 blot force before plunge freezing into liquid ethane and finally transferred to a Titan Krios Transmission Electron Microscope (Thermo Fisher Scientific) operated at 300 keV. Electron micrographs with a calibrated object pixel size of 1.1 Å /pixel were recorded using the EPU software (v 2, Thermo Fisher Scientific, USA) on a K2 direct electron detector (Gatan) operated in counting mode. During data collection, a 20 eV slit was inserted in the Bioquantum 969 energy filter (Gatan), and the zero-loss peak was aligned every two hours. The data acquisition illumination provided an electron flux of 6 electrons/pixel/sec, and the exposure time was accordingly set to provide fluence of 50 electrons/Å^2^ while images were collected in movie-mode and fractionated into 40 frames. All images were recorded at -0.5 μm defocus with a Volta Phase Plate inserted at the back focal plane of the objective lens to improve image contrast. The position of the Volta Phase Plate was set to change every 70 images during data acquisition. On-plane conditions, condenser and objective lens astigmatism, and coma-free alignments were carried out before data acquisition on the carbon area of a Quantifoil grid. Due to severe orientation bias exhibited by the particles, data were collected both at 0° and 30° stage tilt.

### Image processing and 3D reconstructions

**Fig 2:**
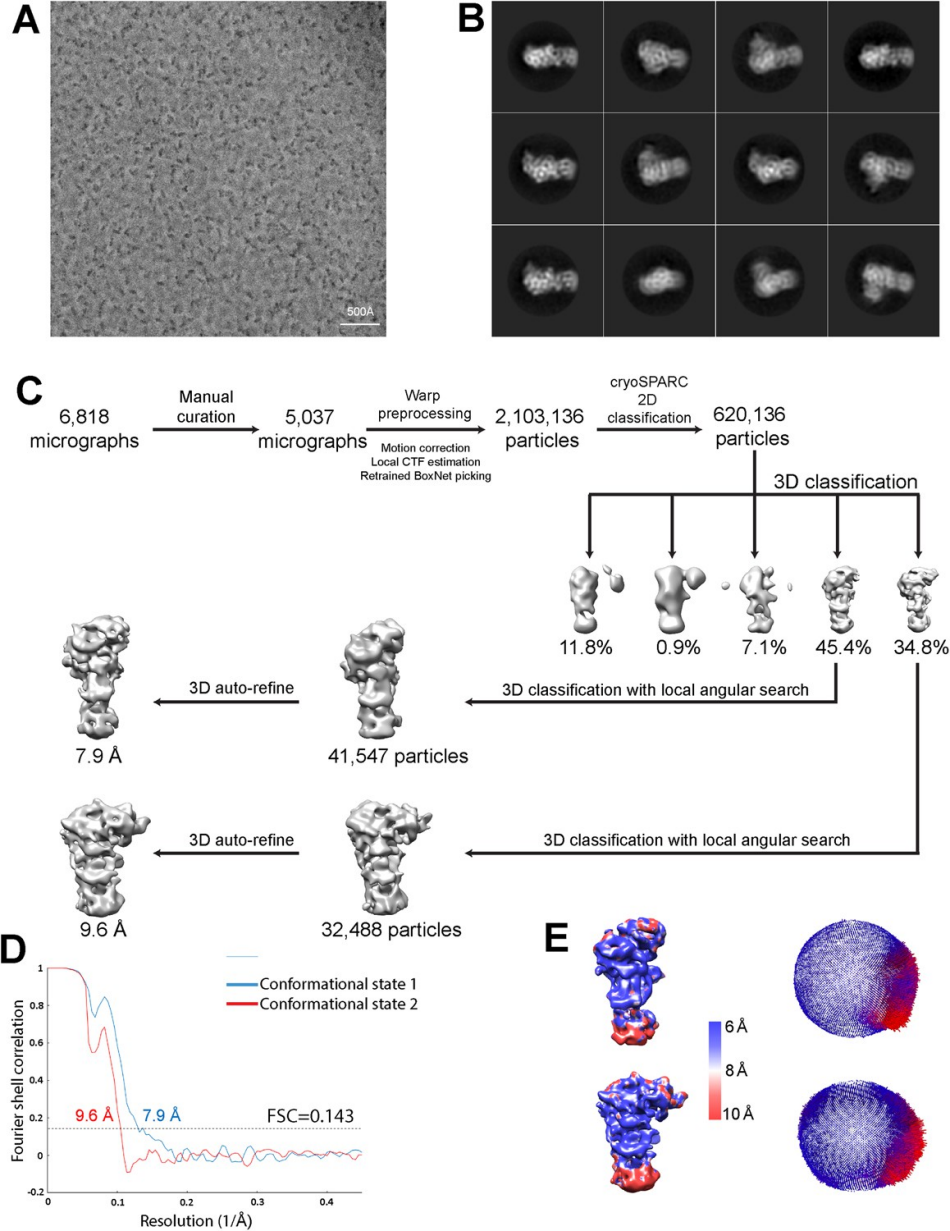
Image processing procedures. (**A**) Representative micrograph. A total of 6,818 micrographs were collected and processed. (**B**) Representative 2D class averages from RELION reference-free 2D classifications. (**C**) Data processing workflow for cryo-EM map reconstruction. (**D**) FSC curves for the two conformations observed, resulting from gold standard 3D refinement. Resolution was determined with a 0.143 FSC threshold. (**E**) Local resolution estimated by Monores and angular distribution analyzed from RELION. Most parts of the two conformational EM structures are resolved in the resolution range of 6–10 Å.

Raw movies for the data from the 0° and 30° stage tilt were combined and pre-processed using Warp’s [36] automated pre-processing pipeline. The combined movies were motion-corrected with a spatial resolution of 5 × 5 for local motion compensation. Contrast transfer function (CTF) parameters were estimated with 9 × 9 spatial resolution for correcting local differences in defocus. Bad micrographs were excluded at this stage based on manual inspection and quality filters, and a curated set of 5,037 micrographs (Fig 2A) was retained. Particles were auto-picked with a retrained BoxNet model based on ~1,000 manually picked particles. A total of 2,103,136 particles were extracted with a box size of 200 × 200 pixels, imported to cryoSPARC v2 [37], and subjected to two rounds of 2D classification (Fig 2B). The selected subset of 620,136 particles was used to generate an initial 3D reference model in cryoSPARC v2, and used for downstream image-processing in RELION. Following the first round of 3D classification with 5 classes inside RELION-3.0 [38], two conformations with a dominant population and distinctive features were observed and selected (Fig 2C) for another round of 3D classification with local angular searches to reduce conformational heterogeneity. A subset 3D class of conformation 1 that consists of 41,547 particles was selected and subjected to a 3D auto-refinement job, which refined to a 7.9 Å resolution EM map (Fig 2C). Likewise, a similar 3D classification and refinement step was performed on conformation 2 and a 9.6 Å resolution reconstruction was obtained from a subset of 32,488 particles (Fig 2C). A soft mask was created for both of the two conformational maps and used for post-processing in RELION. The resolutions reported were calculated based on the gold-standard Fourier shell correlation (FSC) at 0.143 criteria (Fig 2D) [39]. Local resolution was calculated with MonoRes [40] in Scipion [41] with refined half maps and masks created from RELION as input (Fig 2E).

### Electromobility shift assay (EMSA)

Purified recombinant POT1-TPP1-TIN2(1–354) protein complex at concentrations from 100 nM to 10 pM (10^1/3^-fold serial dilution) was incubated with 10 pM 5′-^32^P-labelled telomeric DNA oligonucleotides in a total volume of 22 μl EMSA buffer (50 mM Tris-HCl (pH 8.0), 150 mM KCl, 4 mM MgCl_2_, 0.1% (v/v) Tween-20, 100 μg/ml BSA, 10% (v/v) glycerol, 0.5 ng/μl poly(dI:dC), 1 mM DTT and 1 μM random sequence carrier ssDNA). The reactions were equilibrated at 30° C for 30 min prior to electrophoresis. From each reaction, a 10-μl aliquot of the nucleic acid–protein complex was fractionated (200 V, 35 min) on a pre-electrophoresed non-denaturing (0.5× TB buffer) 4% polyacrylamide gel (37.5:1 mono:bis ratio). Gels were subsequently dried onto a positively charged Hybond N+ nylon membrane (GE Healthcare) and phosphorimaged (Typhoon FLA 7000). The signal intensity of the free DNA was quantified with ImageQuant TL software (Nonlinear Dynamics). The binding affinity (K_d_) of the POT1-TPP1-TIN2(1–354) complex for each DNA oligonucleotide was determined by fitting 14 experimental data points to a four-parameter logistic equation using the Levenberg-Marquardt algorithm:

$$y=F_{min}+\frac{F_{max}K_{d}^{H}}{K_{d}^{H}+x^{H}}$$

Where y is the fraction of unbound DNA at equilibrium, F_min_ is the minimal unbound DNA fraction, F_max_ is the maximal unbound DNA fraction, K_d_ the equilibrium dissociation constant, x is the protein complex concentration at equilibrium and H is the Hill coefficient. Each experiment was repeated at least three independent times (S6 Fig).

### EMSA coupled to Western blot analysis (WEMSA)

The experimental binding conditions of the WEMSA were quasi-identical to the EMSA except that the total concentration of the telomeric DNA oligonucleotide was raised from 10 pM to 10 or 20 nM by supplementing the reactions with unlabeled telomeric DNA of identical sequence. Subsequent to native PAGE fractionation, the nucleic acid–protein complexes were electroblotted (20 V, 120 min, 4° C) onto a PVDF membrane under non-denaturing (0.5× TB) conditions. The PVDF membrane was soaked in 10% acetic acid for 15 min and subsequently air-dried to denature and fix the proteins to the membrane. The nucleic acid–protein complexes were firstly detected by phosphorimaging. In a second step, the proteins bound to the membrane were identified by immunoblotting. Antibody binding was visualised using WesternBright enhanced chemiluminescence HRP substrate (Advanstra) and detected by a CCD camera imager (ChemiDoc Touch, Bio-Rad) (S7A Fig). Tight specificity of the TIN2 (ab197894, Abcam), TPP1 (ab112050, Abcam) and POT1 (ab124784, Abcam) antibodies for their respective antigen and the absence of cross-reactivity with the other components of the complex was verified beforehand (S7B Fig).

### Figure preparation

All Figures showing structures and cryo-EM density maps were generated using UCSF Chimera [42].

## Results

### Cryo-EM analysis

Cryo-EM analyses of the purified and fully assembled shelterin complex (POT1, TPP1, TIN2, TRF2 and RAP1) (see Materials and Methods) were carried out on the unbound complex as well as the shelterin complex bound to a “model” telomere ligand (S1A Fig), containing both the appropriate double and single-stranded binding sites for the DNA-binding shelterin components POT1 and TRF2. However, despite evidence that the shelterin complex was fully active in DNA binding and telomerase activity (S2C Fig), all our original cryo-EM trials produced particles that appeared broken and did not cluster into 2D classes. While we were able to improve particle quality through mild glutaraldehyde crosslinking using the GraFix method [43, 44], 2D classification from large datasets produced only poor 2D classes that did not reconstruct to reliable 3D maps (S4 Fig). Additional efforts in sample and grid optimization did not improve particle homogeneity. Thus, we concluded that the high conformational heterogeneity of the full shelterin complex would prevent structure determination.

On the other hand, cryo-EM analysis of the POT1-TPP1-TIN2(1–354) sub-complex appeared more promising, as our trials using first negative stain and subsequently cryo-EM produced homogeneous monodisperse particles that yielded good 2D classes. However, obtaining cryo-EM data that would allow 3D reconstruction was problematic. Firstly, the particles appeared smaller in size than expected given the molecular weight of the complex (169 kDa) and secondly, they suffered from severe orientation bias impeding 3D reconstructions. Fixation of the POT1-TPP1-TIN2(1–354) complex with crosslinkers or addition of telomeric DNA ligands, would consistently result in substantial degradation of the particle quality, a perplexing effect that could not be countered despite extensive optimization efforts. We hypothesized that the absence of DNA could be exposing a site on the protein complex that promotes sequestration of the particles to the air-water interface. Our hypothesis was supported during cryo-EM screening by the observation that very low concentrations (~0.05 mg/ml) of the POT1-TPP1-TIN2(1–354) sample used to prepare holey EM grids was consistently resulting in very high particle density. Therefore, to counter the effects of a possible adsorption of the particles to the air-water interface, we prepared affinity grids using graphene layered holey grids soaked with pyrene-PEK1K-(Ni)NTA to capture uncleaved POT1-TPP1-TIN2(1–354) complex through its His-10 tag. These grids offered a novel composition of heterogeneous particles of various shapes and sizes (10–20 nm), but did not produce satisfactory 2D classes. We therefore concluded that the otherwise undesirable effect causing our particles to potentially cluster to the air-water interface was actually a useful one, since only under these conditions were we able to obtain satisfactory 2D classes. Thus, we concentrated our efforts on holey UltrAuFoil grids that produced monodisperse POT1-TPP1-TIN2(1–354) particles, but with severe preferred orientation of the complex (Fig 2A).

To overcome the orientation bias and to obtain better 3D information we collected data at a 0° and 30° tilt angle of the electron microscope stage. We also used the Volta Phase Plate (VPP) [45] to improve contrast, because in our initial datasets collected with a tilted stage, the quality of the 2D classes was compromised, most likely due to the increase in ice thickness when imaging a tilted grid. Tilted and un-tilted data sets were combined, and micrographs manually selected for preprocessing. From these micrographs, we extracted 2,103,136 particles for 2D classification (Fig 2B), from which, 620,136 particles were chosen for further 3D classification (Fig 2C). After 3D classification, two predominant and similar classes were present, that represented 45.4% and 34.8% of the total number of particles. These two classes appeared to differ slightly in their shape and were therefore processed and refined independently resulting in the reconstruction of one map (EMD-30596) at 7.9 Å, and the other (EMD-30597) at 9.6 Å resolution. These two maps appeared to correspond to the same particle, but differed in shape. Furthermore, the volume of the maps was not sufficient to accommodate the full POT1-TPP1-TIN2(1–354) complex and hence the two cryo-EM maps represented only part of the complex. In this case, major structural heterogeneity may also be the main cause of the incomplete density maps. Nevertheless, the resulting 3D reconstructions support the existence of (at least) two alternative conformational states of the POT1-TPP1-TIN2(1–354) complex.

### Fitting of the POT1-TPP1-TIN2(1–354) cryo-EM maps

**Fig 3:**
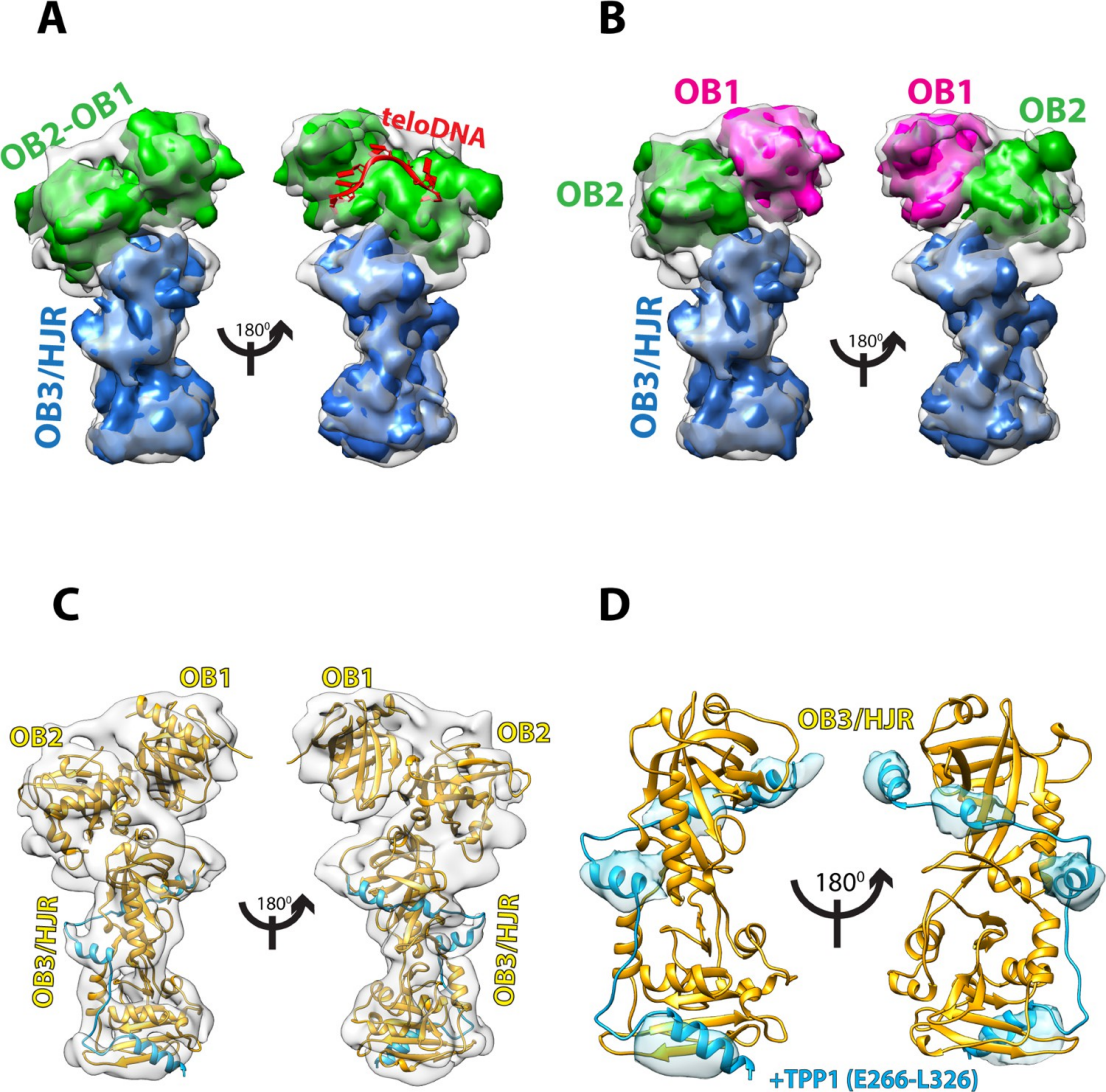
Cryo-EM map at 7.9 Å resolution of POT1 bound by TPP1 (E266-L326) with fitted crystal structures. **(**A**)** Simulated 8 Å maps generated in Chimera for known crystal structures of POT1 domains were fitted into the 7.9 Å cryo-EM map to find structural complementarity. Simulated map for the crystal structure of POT1 (OB1-OB2) (PDB ID: 1XJV) (green) after removal of the ssDNA ligand fits the top half of the map, while simulated map for crystal structure of POT1 (OB3/HJR) complexed to the POT1-binding domain (PBD) of TPP1 (E266-L326) (PDB ID: 5UN7) (blue) fits the bottom half of the map. Placement of ssDNA ligand from PDB ID: 1XJV onto map depicts the location of the DNA-binding groove (red). (**B**) Simulated 8 Å maps for OB1 (magenta) and OB2 (green) demonstrate that improved complementarity of POT1 (OB1-OB2) can be achieved by fitting the OB1 and OB2 domains separately. (**C**) Cartoon representation of the structure of full-length POT1 (yellow) bound by the PBD of TPP1 (blue) which interacts with the OB3/HJR domain of POT1, fitted into the 7.9 Å cryo-EM map. (**D**) Fit of crystal structure of POT1 (OB3/HJR) complexed to the PBD of TPP1 shows that the α-helices of TPP1 (blue) overlap perfectly with four complimentary densities in the map (blue density).

In order to interpret the 7.9 Å cryo-EM map for the POT1-TPP1-TIN2(1–354) complex we used the crystal structures of the various domains of POT1, TPP1, and TIN2 available in the PDB (Fig 1C), and using Chimera [42] we fitted each into the different regions of the map to evaluate complementarity. The fitting of the maps suggests that the OB1, OB2 and OB3/HJR domains of POT1 make up the majority of the density, indicating that the cryo-EM map mostly consists of POT1 with some additional density that corresponds to the PBD of TPP1 (Fig 3).

One half of the cryo-EM 3D-map was fitted with the crystal structure of the DBD of POT1 (OB1 and OB2 folds, residues 1–299) bound to the ssDNA telomeric ligand TTAGGGTTAG (PDB ID: 1XJV) [25]. This provides evidence that this section of the map corresponds to the DBD of POT1 (Fig 3A). Fitting of the crystal structure without removal of the ssDNA ligand pinpoints the DNA-binding site on POT1, which in the cryo-EM map is unoccupied (Fig 3A). As the fit of the two OB folds into the map was suboptimal, the crystal structure was segmented and each OB fold fitted independently. This improved the fit for both, especially for OB1 (Fig 3B). This provides evidence that the relative orientation or conformation of the two OB folds (OB1 and OB2) in our cryo-EM map is somewhat different from that observed in the crystal structure of the two POT1 OB folds bound to the telomeric ssDNA ligand (S5A Fig).

The other half of the EM map could be fitted with high complementarity with the crystal structure of the C-terminal half of POT1 (OB3/HJR, residues 332–633) bound by the POT1-binding domain (PBD) of TPP1 (E266-L326) (PDB ID: 5UN7, 5H65) [23, 24] (Fig 3). Furthermore, the four α-helices of the PBD of TPP1 overlaid optimally with four corresponding densities in the map, indicating the PBD region of TPP1 is present and is bound to POT1 exactly as described in two published identical crystal structures (PDB ID: 5UN7, 5H65) [23, 24] (Fig 3D). Interestingly, there is no apparent density for the rest of TPP1. As the regions connecting the TPP1 domains are long and unstructured [15], it is likely the TPP1 domains are not held into one rigid globular conformation, but could be rather flexible and elude 2D classification. Alternatively, this could also be due to TPP1 denaturing during the blotting and vitrification process, while the PBD remained protected by binding to POT1.

In our cryo-EM map there is no additional density to account for TIN2, despite its presence in the complex prior to EM grid preparation. There could be several reasons for the lack of density: Similarly to TPP1, TIN2 could be quite dynamic in relation to POT1 and its presence lost during 2D classification, or it may have also been denatured during grid preparation. Finally, since our POT1-TPP1-TIN2(1–354) complex was not crosslinked, another possibility is that the TIN2(1–354) component detached from the POT1-TPP1 complex during grid preparation.

**Fig 4:**
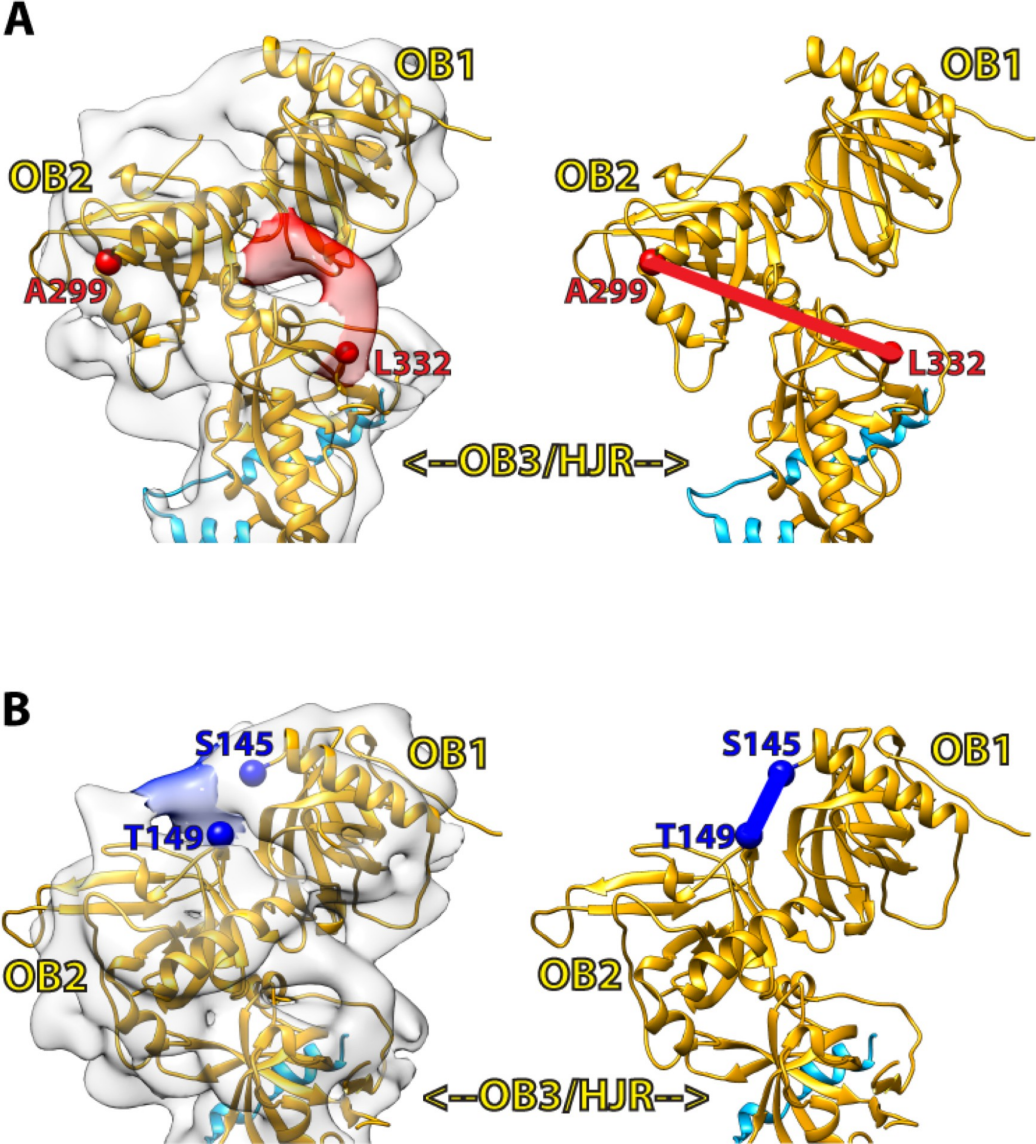
Unoccupied densities in the map provide insights into unresolved regions. (**A**) An unoccupied density (red density) at the POT1 OB2-OB3/HJR junction extends from where L332 (red sphere) terminates and could possibly account for part of the linker region bridging L322 (red sphere) of OB3/HJR to A299 (red sphere) of OB2. (**B**) An unoccupied density (blue density) extends from S145 (blue sphere) in OB1 and into OB2, but does not overlay with where the linker region connecting OB1 to OB2 (S145-T149, blue line) is located in the crystal structure (PDB ID: 1XJV).

Currently, there is no structural information on the 32-amino acid linker region connecting L332 from the C-terminal half of POT1 to A299 of the OB2 domain. In our fitted cryo-EM map, some unoccupied densities that surround the region where OB2 meets OB3/HJR are visible, and could therefore account for the missing segment linking OB2 to OB3/HJR, or possibly correspond to additional parts of TPP1 as well. One noteworthy unresolved density appears to extend beyond L332 and towards the OB2 fold (Fig 4A). Since this region of the map contains some of the higher resolution information of the reconstruction (Fig 2E), the unresolved density is likely not an artefact, and may correspond to part of the 32-amino acid linker region. However, the resolution of our map limits a more precise interpretation and multiple rounds of fitting with Molecular Dynamics Flexibility Fitting (MDFF) [46] failed to provide additional insights. Another noteworthy density appears to bridge OB1 to the OB2 (Fig 4B), but does not overlay with the linker connecting OB1 to OB2 in the crystal structure. A likely interpretation is that the conformation and orientation of OB2 and/or the linker connecting OB1 to OB2 in full-length and DNA-free POT1 is significantly different to the one observed in the crystal structure. This suggestion is supported by the less than optimal fit of OB2 in comparison to that of the OB1 and OB3/HJR domains. However, in the absence of a higher resolution cryo-EM map of full-length POT1 in complex with its DNA ligand, the cause of this difference remains elusive.

### Two conformations for the DNA-binding domain of POT1

Because the available structural information on POT1 is limited to the X-ray structures of the two halves [DNA-binding domain (OB1-OB2) and TPP1-binding domain (OB3/HJR)] [23–25] of POT1, the model derived from our cryo-EM analysis provides the first view of the structure of full-length POT1. In full-length POT1, the N-terminal OB1-OB2 half pivots from the OB3/HJR domain at a ~45° angle (Fig 3B and 3C). Whilst the crystal structure of OB3/HJR bound by the PBD of TPP1 fits well into the cryo-EM map (Fig 3), the improved fit of the OB1 and OB2 domains when fitted separately suggests that full-length POT1 can adopt different conformations, particularly in the relative orientation of the OB folds when not bound to DNA (Fig 3B). OB1 and OB2 are connected by a short linker (S145-T149), and in the crystal structure of POT1 bound to DNA, the middle three amino acids (P146-W148) were unresolved, suggesting some flexibility. In the cryo-EM map, the unresolved density connecting OB1 to OB2 (Fig 4B) also suggests additional flexibility in that region. Thus, this flexible linker could potentially allow OB1 and OB2 to take up a tighter conformation when bound to DNA.

**Fig 5:**
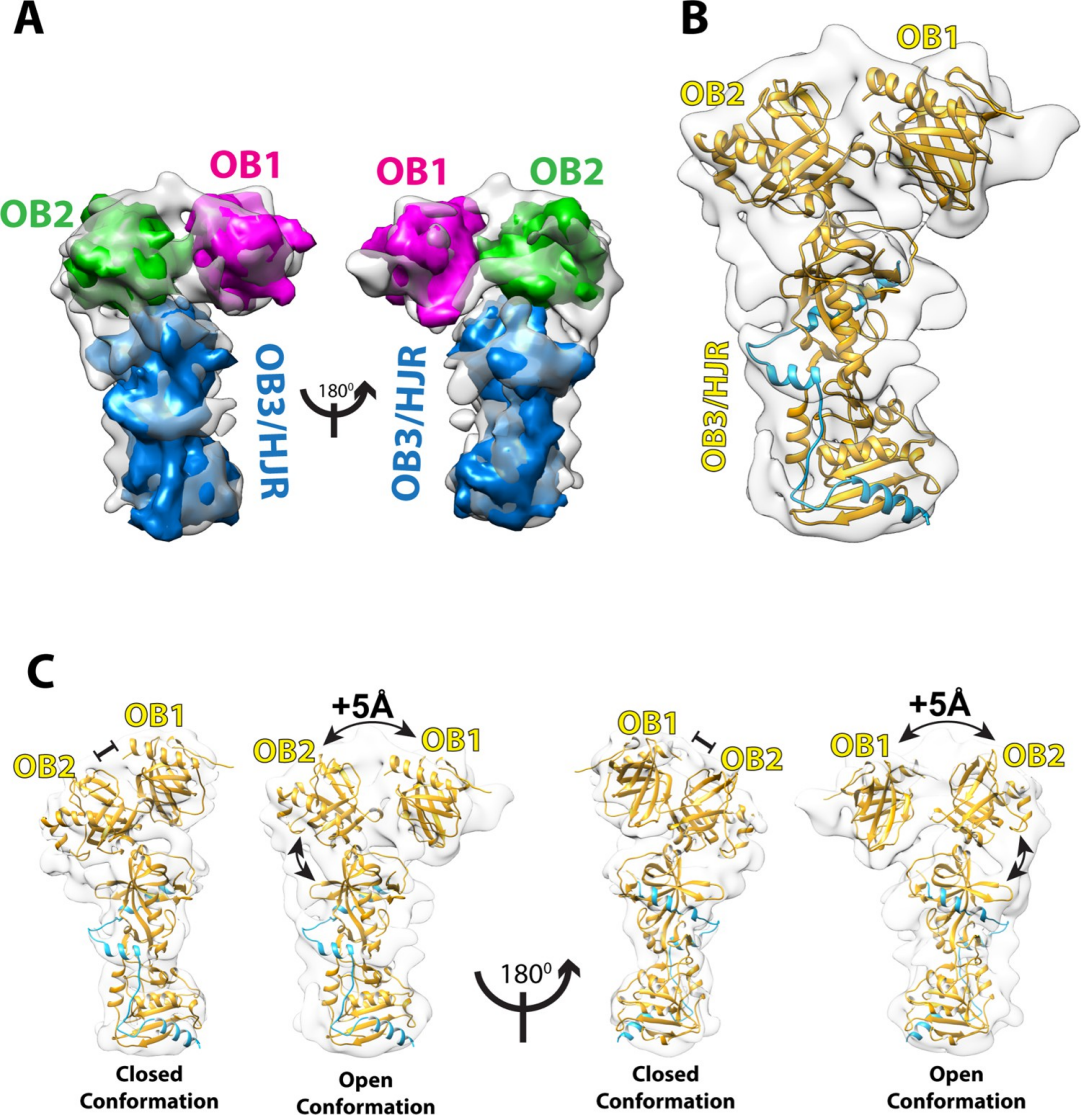
Cryo-EM map at 9.6 Å resolution of POT1 with fitted crystal structures indicates an alternative open conformation. (**A**) Simulated 8 Å maps generated with Chimera for POT1 OB1 (magenta) and OB2 (green) fitted into the 9.6 Å cryo-EM map suggest the two domains exist in an open/extended conformation. (**B**) Cartoon representation of full-length POT1 (yellow) bound by the PBD of TPP1 (blue) fitted into the 9.6 Å cryo-EM map. (**C**) Comparison between closed and open conformations of full-length POT1 complexed to the PBD of TPP1 observed through cryo-EM. The distance between the centres of OB1 and OB2 is increased by about 5 Å in the open conformation compared to the closed conformation.

The second reconstruction obtained from the same dataset for the POT1-TPP1-TIN2(1–354) complex provides additional insights. The resolution of this map is 9.6 Å, and therefore more difficult to interpret *de novo*. However, by making use of the fitting of structures into the 7.9 Å map, we were able to place each of the POT1 domains (OB1, OB2, and OB3/HJR) into the 9.6 Å map in similar relative orientations (Fig 5A). While it is impossible to discern some of the details that were apparent in the 7.9 Å map, what is clearly evident is that whilst most of the overall density of the 9.6 Å map overlays well with the 7.9 Å map, the relative orientations of the OB1 and OB2 folds are distinctively different to what was observed in the 7.9 Å map, or in the crystal structure of the POT1 OB1 and OB2 domains bound to the ssDNA ligand (S5B and S5C Fig). While in the 7.9 Å map, OB1 and OB2 take up a compact or closed conformation that resembles that seen in the crystal structure of the OB folds bound to DNA (closed conformation, Fig 5C), in the 9.6 Å map the density for OB1 is displaced relative to OB2 taking up an extended conformation (open conformation, Figs 5C and S5C). The global movement, measured as the centre-to-centre distance between the two OB folds (as defined from fitting the two globular domains into the two EM maps), increases by about 5 Å in the open state (Fig 5C). Furthermore, the relative orientation of the OB2 domain as it extends out of the OB3/HJR domain also appears to differ in the two maps, suggesting that the OB2 domain may also swing. This observation suggests that POT1 has a dynamic structure in which the three domains (OB1, OB2, and OB3/HJR) are flexibly linked (Fig 5B and 5C) resulting in alternative conformations of full length POT1 (S5 Fig).

In summary, we have been able to fit most of the two cryo-EM density maps that dominate the family of reconstructions for the POT1-TPP1-TIN2(1–354) complex. The fitting of the known structures of sub-domains into the 7.9 Å map demonstrates that the reconstruction we have obtained represents full-length POT1 bound by the PBD of TPP1. Comparison to the 9.6 Å map reveals a different conformation for the DBD of POT1 in which the two OB folds have moved apart and are in a more open conformation than the closed one observed in our 7.9 Å map. The alternative conformations of OB1 relative to OB2 indicates a flexibility in the DBD of POT1 that likely has implications for how POT1 binds to telomeric DNA repeats.

### DNA binding studies suggest flexibility in DNA binding by POT1

**Fig 6:**
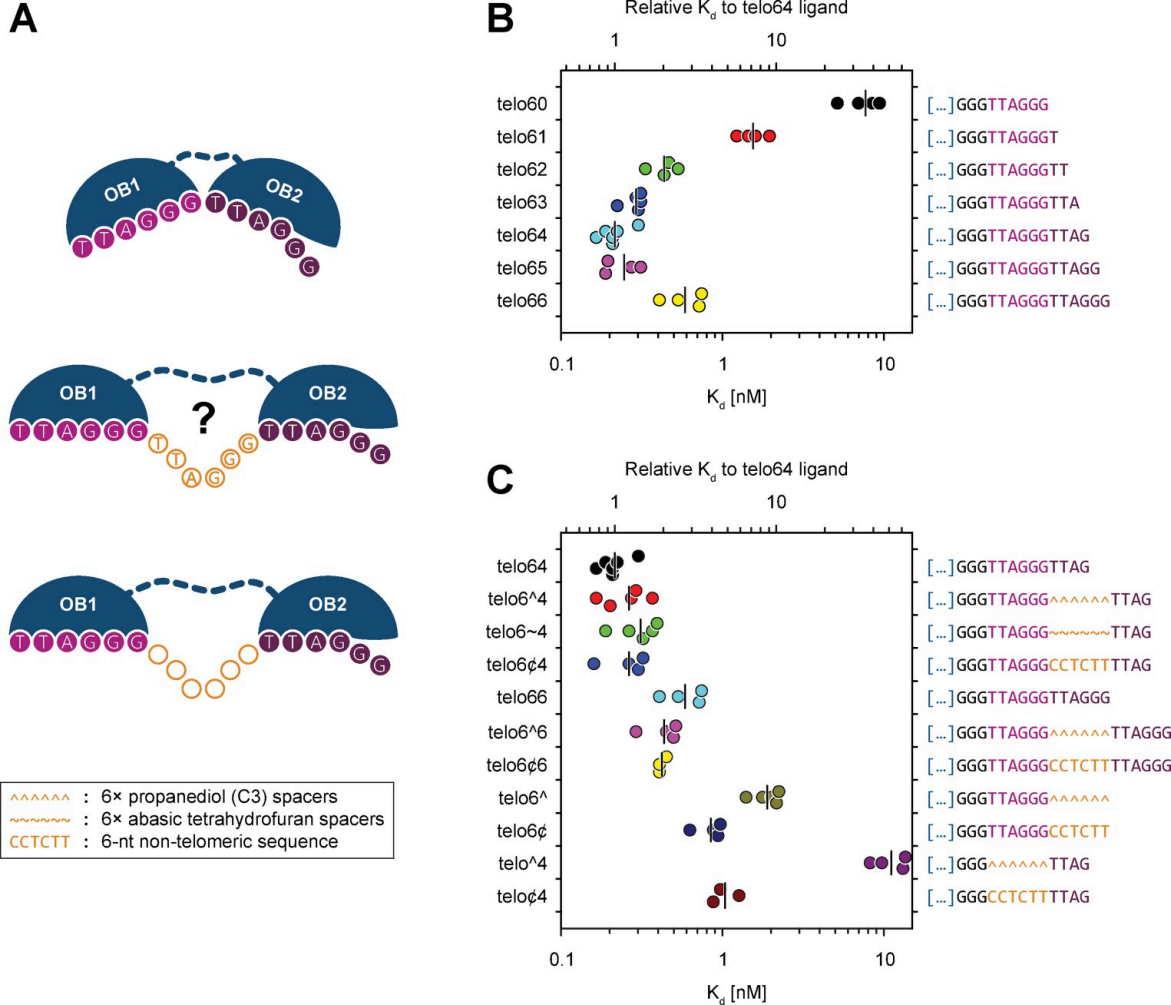
EMSA demonstrate that binding of telomeric ssDNA to the POT1 OB1/OB2 domain is not limited to the minimal binding sequence, but to sequences separated by spacers as well. (**A**) Cartoon representation of the POT1 OB1/2 domains and its possible binding conformations that can allow binding to the minimal tight binding sequence TTAGGGTTAG (top), or the same sequence separated by either a single telomeric repeat (center) or a ~6-nt long non-telomeric spacer (bottom). (**B**) Horizontal scatter plot with median marking of the apparent binding affinity of various telomeric ligands for the POT1-TPP1-TIN2(1–354) subcomplex. Each individual K_d_ value was computed from the EMSA data shown in S6B–S6H Fig. The binding experiments confirm that telo64 is truly the minimal tight-binding sequence. Both shorter and longer oligos, with the notable exception of telo65 ligand, present reduced affinities for the subcomplex. (**C**) Horizontal scatter plot with median marking of the apparent binding affinity of various non-contiguous telomeric ligands for the POT1-TPP1-TIN2(1–354) subcomplex. Each individual K_d_ value was computed from the EMSA data shown in S6I–S6Q Fig. The binding experiments show that the minimal tight-binding sequence ligands that have been interrupted with various non-telomeric spacers retain binding affinity comparable to that of the contiguous telo64 ligand.

Having concluded from the structural information that the DBD of POT1 may exist in an open and closed conformation, we next addressed the question of how such flexibility may affect DNA binding. From the crystal structure of the DBD of POT1 bound to the minimal ssDNA telomeric sequence TTAGGGTTAG we know that it is a rather compact structure in which the two OB folds pack together providing a continuous binding cavity: OB1 binds to a half-site consisting of the first six nucleotides (TTAGGG) of the binding site, while OB2 binds to a half-site consisting of the adjacent four nucleotides (TTAG) [25]. Interestingly, the relative orientation of the two OB folds in the crystal structure introduces a bend in the cavity where the two domains meet, creating a kink in the DNA phosphodiester group of dT7 which causes a 90° change in the directionality of the DNA strand (Fig 1C) [25]. Therefore, based on these observations, we decided to reinvestigate the DNA binding properties of the POT1-TPP1-TIN2(1–354) complex in respect to previously published data for POT1 alone [25, 47]. To assess the role of the conformational flexibility observed, binding experiments were designed using model telomeres in which the G-overhang consisted of shorter and longer constructs of the minimal high affinity POT1 binding sequence TTAGGGTTAG and also constructs where the two half-sites of the OB folds were separated by a spacer (S1 Fig, Fig 6A).

As already observed for POT1 alone [25, 47], truncation of the 3′ end of the minimal tight binding telomeric DNA ligand caused a reduction in binding affinity (Figs 6B and **S6B–S6F**). Specifically, while the minimal tight binding sequence TTAGGGTTAG (telo64) demonstrated the highest affinity, each nucleotide removed from the 3′ end caused a progressive reduction in affinity with TTAGGG (telo60) showing the least binding affinity. On the other hand, extending the previously reported minimal tight binding sequence by a single 3′ dG (telo65) did not significantly change the binding affinity of the complex, but extending it by two dG’s (telo66) decreased the affinity (Figs 6B and **S6F–S6H**). Therefore, the results of our binding experiments for the POT1-TPP1-TIN2(1–354) complex corroborate previous results obtained with POT1 alone [25, 47], confirming that TTAGGGTTAG is indeed the minimal tight binding sequence for POT1 and consistent with binding via a closed conformation as seen in the crystal structure of the ligand-bound OB folds [25].

Having established that the POT1-TPP1-TIN2(1–354) complex has similar binding properties as previously reported for POT1 alone, we went on to investigate whether the open conformation seen for the two POT1 OB folds in the second cryo-EM map could potentially represent a biologically relevant conformation. We hypothesized that it might be possible for POT1 to bind telomeric DNA by recognizing repeats that are not adjacent to each other, but spaced apart as is possible in the tandemly arranged telomeric repeats. To corroborate this hypothesis, we designed telomeric binding sites for POT1 in which an approximate six-nucleotide-long unnatural DNA spacer (corresponding to the length of a single telomeric repeat) had been introduced between the half-sites for OB1 and OB2 (S1B Fig). Since POT1 binds its telomeric ligand through the base moieties on the DNA, we resorted to introduce two types of spacers, each including an hexad of abasic phosphoramidites. The spacer made of the propanediol (C3) phosphoramidites lacks the furanose ring but retains the C3, C4 and C5 carbons of 2-deoxyribose which compose the phosphodiester backbone. On the other hand, the spacer consisting of the tetrahydrofuran analogue phosphoramidite is structurally closer to a natural apurinic/apyrimidinic site. In addition, a non-telomeric sequence of six nucleotides in length was also introduced to ascertain whether incorporation of native non-telomeric DNA would affect the binding affinity (Figs 6A and S1B).

The results of our DNA binding studies (Figs 6C and **S6I–S6Q**) show that oligonucleotide constructs that contain a single telomeric repeat (the half-site for OB1) and the C3 spacer (telo6^, S6N Fig), bound the POT1-TPP1-TIN2(1–354) complex with an affinity comparable to that containing a single telomeric repeat (telo60, S6B Fig), or a single telomeric repeat with an extra dT on the 3′ end (telo61, S6C Fig). This result provides evidence that the POT1-TPP1-TIN2(1–354) complex does not bind to the abasic C3 spacer (Figs 6C and S6N). In contrast, it was possible to nearly recover the high binding affinity displayed by the minimal tight-binding sequence ligand (telo64, S6F Fig) by extending the telo6^ telomeric binding site with TTAG (half-site that binds OB2) on the 3′ side of the abasic spacer (telo6^4, Figs 6C and S6I). DNA ligands containing the acyclic C3 spacer are likely to be more conformationally flexible than those containing the tetrahydrofuran analogue spacer. Nevertheless, the relative conformational flexibility of the spacer does not seem to be relevant as similar binding affinity was observed when the C3 spacer was substituted with the tetrahydrofuran analogue linker (telo6~4, S6J Fig), or for the non-telomeric CCTCTT spacer (telo6¢4, Figs 6C and S6K). Introducing the 6-nt long non-telomeric spacer reduced the affinity only marginally (by a factor of 1.4 to 1.7), compared to that of the minimal tight-binding sequence ligand (telo64, Fig 6C).

In summary, our DNA binding studies using a variety of telomeric binding ligands demonstrate that POT1 can indeed bind alternative telomeric DNA sequences in which the half-sites are spaced apart, and is not strictly confined to binding to telomeric repeats adjacent to each other. These results suggest that the open and closed conformation of the DNA binding OB folds of POT1 observed in our cryo-EM analysis increases its DNA binding possibilities, which has implications for how the tandemly arranged binding sites are recognized by telomeric proteins.

## Discussion

POT1 is a shelterin component essential for telomere maintenance [48]. It binds to the telomeric G-overhang and together with its interacting partner TPP1 helps to recruit telomerase to telomeres, preventing severe telomere instability [21]. Here we report two cryo-EM maps that correspond to full-length human POT1 in complex with the POT1-binding domain (PBD) of TPP1 (E266-L326). Comparison of the maps reveal an open and closed conformation for the first two OB folds that constitute the DNA-binding domain (DBD) of POT1. Furthermore, our DNA binding studies reveal that POT1 can bind to telomeric sequences in which the binding sites for the OB1 and OB2 domains are spaced apart, suggesting that POT1 can bind to non-continuous telomeric binding sites and hence has plasticity in binding.

It was disappointing that our cryo-EM reconstructions show density for only part of the POT1-TPP1-TIN2(1–354) complex. Given that all telomeric proteins contain unstructured regions, the most likely reason for the missing density is that conformational flexibility prevents particle alignment and hence 2D classification required for 3D reconstruction by cryo-EM. Alternatively, shelterin complexes are simply unstable under cryo-EM conditions. On the other hand, POT1 in complex with TPP1 appears to be sufficiently rigid to permit 2D classification, resulting in two very similar and well-populated 3D-reconstructions that significantly differ in the relative orientation of the two DNA-binding OB folds of POT1, indicating an open and closed conformation for the DBD of POT1.

Could this structural flexibility observed for the DBD of POT1 affect telomeric DNA recognition? Because of the length of the telomeric G-overhang and the tandem arrangement of telomeric repeats, telomeric single-strand binding proteins encounter an abundance of closely spaced binding sites and hence multiple choices or opportunities for sequence-specific binding. This is unlike the situation for transcription factors that typically have defined binding sites specified by specific DNA sequences embedded in the genome. POT1 aligns on the telomeric G-overhang recognizing the telomeric decamer sequence TTAGGGTTAG. Whilst in the crystal structure of the complex between the telomeric decamer sequence and the DBD of POT1, the two OB folds pack closely together forming a continuous binding surface, they each recognize a “half-site”: the OB1 domain binds the first six nucleotides TTAGGG (that corresponds to a telomeric repeat), while the adjacent OB2 domain with the last four, TTAG (or the first four nucleotides of the adjacent telomeric repeat). Since our binding experiments show that the two “half-sites” can be spaced apart without a loss in DNA binding affinity (Fig 6C), we can conclude that there is also a degree of flexibility in DNA binding. The alternative or open conformation, of the two POT1 OB folds could be permitted by the five amino acid stretch (S145–T149) linking the two OB folds, that is partly unstructured in the crystal structure of the POT1 OB folds in complex with the decamer telomeric DNA ligand [25]. For the telomeric ligand, the length of the spacers we introduced between the two binding “half-sites” is six nucleotides in length and hence corresponds to the length of a telomeric repeat. An interpretation of the DNA binding experiments together with the structural information from our cryo-EM structures is that POT1 is not restricted to binding adjacent telomeric repeats but also telomeric repeats spaced apart. This plasticity in binding could be advantageous in ensuring G-overhang protection even at low levels of POT1.

Additionally, the plasticity of the OB folds in DNA binding may also have implications in G-quadruplex unfolding. Studies have demonstrated POT1 can resolve intramolecular G-quadruplexes, secondary structures formed by guanine-rich sequences through cyclic Hoogsteen base pairing, that stall telomerase when formed [49]. POT1 has been reported to be able to restore telomerase processivity by resolving these G-quadruplexes and thus freeing the 3’-end of the G-overhang for telomerase to work on [49]. But while several models on how POT1 interacts and unfolds G-quadruplexes have been proposed, OB fold flexibility as a possible parameter has not been discussed in the literature [49–52].

The flexible behavior we have observed for the OB folds bears similarities to that of POT1 in *Schizosaccharomyces pombe*, which is structurally divergent from human POT1 but retains a comparable domain topology [53]. In *S*. *pombe* POT1 however, the DBD binds a 15-nt sequence as a monomer, and a shorter 12-nt sequence as a dimer, a behavior not observed in human POT1 [53]. Also, unlike human POT1, in *S*. *pombe* each of the two OB folds of the DBD have distinct and independent binding modes, and are connected by a long linker, while a transient and subtle intermolecular interface between the two domains appears to form only when bound to DNA [53]. Similarly, it is therefore also likely that in human POT1 the OB fold flexibility may improve efficiency of binding to tandem repeats, while a more rigid conformation may form when bound to an optimal sequence, such as the telomeric 3’ end.

To some degree, the binding mode of POT1 is also reminiscent to that of TRF1 and TRF2 that bind to the double-stranded telomeric repeats and hence also encounter a high concentration of closely spaced binding-sites. TRF1 and TRF2 also have a flexible architecture. They both function as homodimers in which the two DNA binding domains are linked to the dimerization domain by very long unstructured linkers [29, 30]. For TRF1, experimental binding data showed that it can bind with the same affinity and specificity to bipartite telomeric sites with extreme spatial flexibility [54]. It therefore appears that proteins that bind to telomeric DNA have DNA-binding domains that consist of two binding modules whose conformation is spatially adjustable. This flexibility may aid in the efficient recognition of tandemly arranged binding sites that present multiple opportunities for binding. An explanation for the intrinsic flexibility in binding by DNA-binding domains consisting of two DNA-binding modules could be to ensure that telomeric factors can stay bound to telomeres whilst releasing half-sites during transcription, replication or telomere synthesis, and thus ensuring telomere protection.

## Supporting information

S1 Fig Secondary structure and list of DNA sequences used in this work. (A) Schematic representation of the prototypical shelterin ligand used in this work. Nearly all DNA ligands comprise both single-stranded and double-stranded telomeric regions with a 5′ 5-nt-long non-telomeric double-stranded extremity to ensure correct strand hybridisation register with the antisense DNA oligo ctelo. A 15-bp-long telomeric sequence enables the binding of TRF2 onto the 5′ side of the G-rich strand while a 10-to-12-nt-long single-stranded 3′ telomeric overhang constitutes the binding site for POT1. TRF2 and POT1 binding sequences are spaced by a 3-nt-long single-stranded telomeric sequence to limit steric hindrance between POT1 and TRF2. (B) Inventory and sequences of the DNA ligands used in this work. Double-stranded regions are underlined. The telo666 oligo used for the telomerase activity assay carries a G-to-C mutation (depicted as a lower-case letter) to prevent POT1 binding the 3′-extremity of the G-rich strand and to compete with telomerase binding [55]. The various type of spacer sequences are located between the OB1 and the OB2 site of POT1 and are depicted in orange. Spacer sequences consist of six residues of either: a C3 propanediol spacer (^), an abasic tetrahydrofuran (1′,2′-dideoxyribose) spacer (~) or a non-telomeric hexad (CCTCTT) sequence.
(PDF)

S2 Fig Purity and biochemical activity analysis of the purified recombinant shelterin complex and subcomplexes. (A) Purity of shelterin subcomplexes POT1-TPP1-TIN2(1–354) (left) and TRF2-RAP1 (right) was assessed by SDS-PAGE and Coomassie staining and found to be >95% pure. The dotted black vertical line in the POT1-TPP1-TIN2(1–354) gel depicts splicing of image in order to remove irrelevant lanes (B) Analytical SEC chromatograms of POT1-TPP1-TIN2(1–354) (green), TRF2-RAP1 (red), and reconstituted shelterin (POT1-TPP1-TIN2(1–354) + (2×)TRF2-RAP1, yellow). (C) Direct telomerase assay to assess the biological activity of the purified recombinant POT1-TPP1-TIN2(1–354) complex on the enzymatic activity of the human telomerase. The double-stranded telomeric ligand (telo666) was pre-incubated alone (−) or with increasing quantities (5 to 100 nM, 20^1/7^-fold serial dilution) of fully (POT1-TPP1-TIN2(1–354)-TRF2-RAP1) or partially (POT1-TPP1-TIN2(1–354) or TRF2-RAP1) reconstituted shelterin sub-complexes prior to proceeding with telomerase-catalysed primer extension. The reaction products were analyzed by denaturing PAGE and phosphorimaged. Telomeric repeats are indicated with black diamonds. The position of the 1-, 10-, 20- and 30-fold repeats is depicted as twin diamonds. Results show that when present in the reaction, the purified recombinant POT1-TPP1-TIN2(1–354) complex alone or when associated with TRF2-RAP1 increases stoichiometrically the telomerase processivity. As a control, TRF2-RAP1 alone does not alter substantially the telomerase processivity.
(TIF)

S3 Fig Analytical Size Exclusion Chromatography of the POT1-TPP1-TIN2(1–354) complex bound to a single-stranded telomeric substrate **GGTTAGGGTTAG** (sstelo64). Straight lines represent the 280 nm absorbance and dotted lines represent the 260 nm absorbance. The blue traces represent the elution profile of an injection of 600 pmol (1×) POT1-TPP1-TIN2(1–354). The red traces represent the elution profile of an injection of 600 pmol (1×) POT1-TPP1-TIN2(1–354) incubated with 1200 pmol (2×) sstelo64 ssDNA ligand. The green traces represent the elution profile of an injection of 600 pmol (1×) sstelo64 ssDNA ligand. The black traces represent the elution profile of an injection of 1200 pmol (2×) sstelo64 ssDNA ligand. Comparison of the elution profiles are depicted as follows: (A) protein(1×)+ssDNA(2×) vs ssDNA(2×). (B) protein(1×)+ssDNA(2×) vs ssDNA(1×). (C) protein(1×) vs ssDNA(1×). (D) protein(1×)+ssDNA(2×) vs protein(1×). Comparison of the various elution profiles show that the POT1-TPP1-TIN2(1–354) complex binds approximately an equimolar quantity of its ssDNA ligand indicating a ~1:1 binding stoichiometry.
(TIF)

S4 Fig 2D classes from cryo-EM and negative stain EM for the fully assembled shelterin complex. (A) Top 30 2D classes from cryo-EM of the fully assembled shelterin complex shows classes that are heterogenous and do not reconstruct into a reliable 3D map. (B) Top 32 2D classes from negative stain EM of the fully assembled shelterin complex shows similar representation of classes as in cryo-EM, which also suggests there is high conformational heterogeneity in the sample.
(TIFF)

S5 Fig Superimposition of POT1 structures on the OB1 domain. (A) Closed conformation of TPP1(blue)-bound POT1(yellow) structure superimposed to the X-ray structure (PDB ID: 1XJV) of the DNA(red)-bound POT1(green) OB1 and OB2 domains by aligning on the OB1 domain. The comparison between the two structures shows that the OB2 domains do not properly superimpose and that the OB2 domain in the DNA-bound crystal structure is sterically clashing with the OB3/HJR domain suggesting an alternative conformation likely forms in full-length POT1. (B) Open conformation of TPP1(blue)-bound POT1(yellow) structure superimposed to the X-ray structure (PDB ID: 1XJV) of the DNA(red)-bound POT1(green) OB1 and OB2 domains by aligning on the OB1 domain. The comparison between the two structures shows that the OB1 and OB2 domains are spaced apart in the open conformation resulting in a significant conformational difference between the two structures. (C) Superimposition on the OB1 domain of the closed (yellow) and open (blue) conformations of full-length POT1 complexed to the PBD of TPP1 observed through cryo-EM shows a significant difference in the position of the OB2 and OB3/HJR domains relative to the OB1 domain.
(TIF)

S6 Fig EMSA analysis of the binding affinity of the POT1-TPP1-TIN2(1–354) complex for various telomeric sequence constructs. The various ^32^P-labelled telomeric DNAs were incubated alone (−) or with increasing quantities (10 pM to 100 nM) of POT1-TPP1-TIN2(1–354) protein complex. The binding reaction products were separated by native PAGE. The radio-labelled telomeric DNA was detected by phosphorimaging. The filled and open arrows on the left denote the positions of the retarded protein-DNA complex and the free DNA, respectively. For each telomeric DNA construct investigated, a representative EMSA image is shown (upper panel) and quantification data of the unbound DNA fraction is depicted (lower panel) for all the independent experiments. K_d_ values were determined by non-linear regression and are shown in Fig 6B and 6C. The sequence of the single-stranded fragment of each ligand is depicted on the top of the panel and the complete sequence is available in S1B Fig. Investigated telomeric ligands are: (A) telo00, (B) telo60, (C) telo61, (D) telo62, (E) telo63, (F) telo64, (G) telo65, (H) telo66, (I) telo6^4, (J) telo6~4, (K) telo6¢4, (L) telo6^6, (M) telo6¢6, (N) telo6^, (O) telo6¢, (P) telo^4 and (Q) telo¢4.
(PDF)

S7 Fig Confirmation of the composition of the POT1-TPP1-TIN2(1–354) complex by immunoblotting analysis. (A) POT1-TPP1-TIN2(1–354) complex integrity analysis by WEMSA. ^32^P-labelled telo64 telomeric DNA ligand at a concentration of 20 nM (upper panel) or 10 nM (middle and lower panels) was incubated alone (−) or with increasing quantities (10 pM to 100 nM) of POT1-TPP1-TIN2(1–354) protein complex. The binding reaction products were separated by native PAGE and electroblotted onto a PVDF membrane. The radio-labelled telomeric DNA was detected by phosphorimaging (left panels). The simultaneous presence of TIN2(1–354), TPP1 and POT1 proteins with the retarded mobility DNA bands was verified by probing the membrane with either anti-TIN2 (upper-middle panel), anti-TPP1 (central panel) or anti-POT1 (lower-middle panel) antibodies. Coincidence analysis of the ECL and ^32^P signals was obtained by stacking the phosphorimaging and immunoblotting data using the membrane corners as alignment references (right panels). The filled and open arrows denote the positions of the retarded protein-DNA complex and the free DNA, respectively. Data are representative of three independent experiments. (B) Western-blot analysis of the binding specificity of the anti-TIN2, anti-TPP1 and anti-POT1 antibodies. Proteins from 10-μl aliquots of the binding reactions shown in panel (A) were separated by SDS-PAGE, transferred onto a PVDF membrane and sequentially probed with anti-TIN2, anti-TPP1 and anti-POT1 antibodies. Positions and sizes (kDa) of marker proteins are shown at the left.
(PDF)
