# Supplementary figures and images for: Insights into POT1 structural dynamics revealed by cryo-EM
Source: PLoS One. 2022 Feb 17;17(2):e0264073. doi: 10.1371/journal.pone.0264073 (PMC8853558; doi:10.1371/journal.pone.0264073)

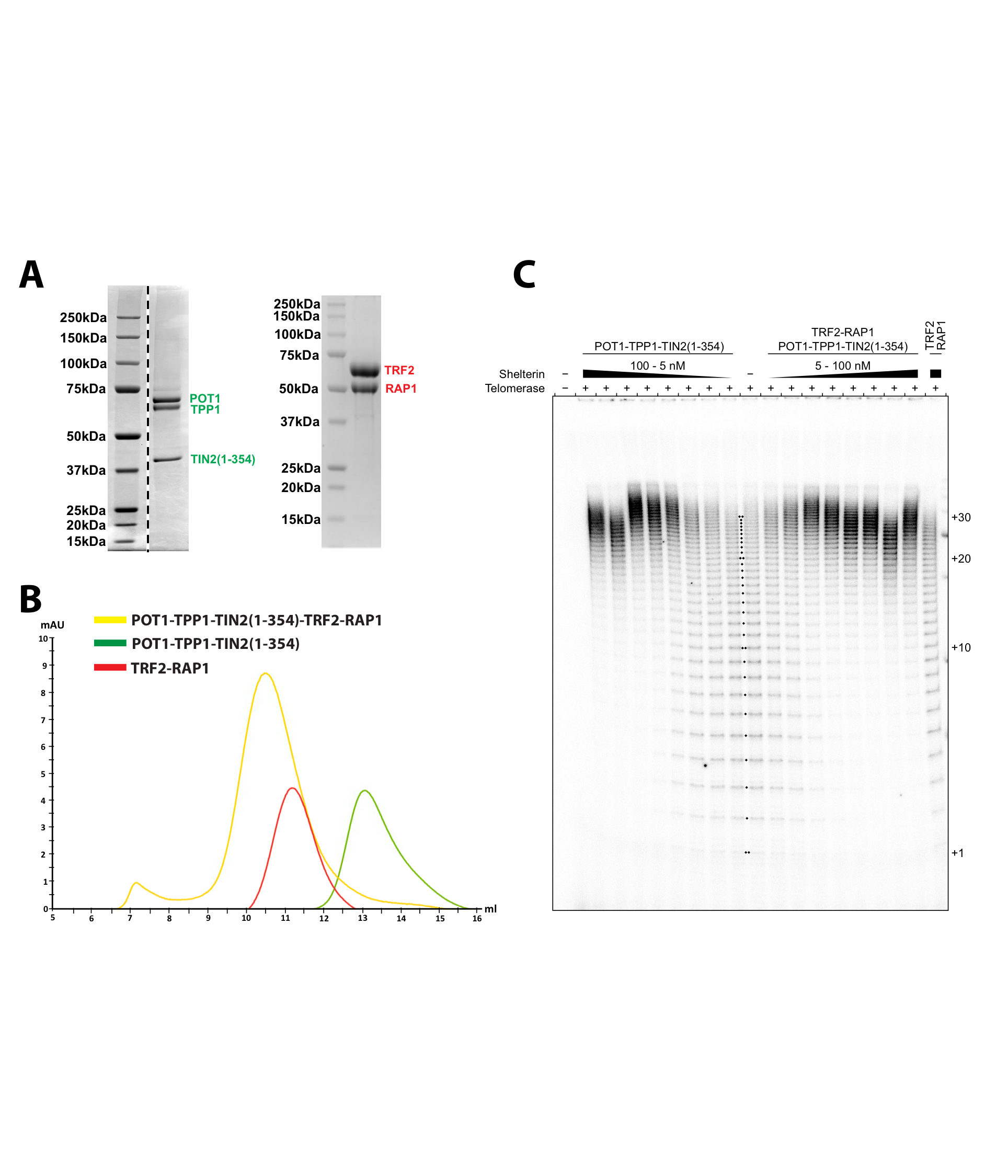

Supplement: S2 Fig — (A) Purity of shelterin subcomplexes POT1-TPP1-TIN2(1–354) (left) and TRF2-RAP1 (right) was assessed by SDS-PAGE and Coomassie staining and found to be >95% pure. The dotted black vertical line in the POT1-TPP1-TIN2(1–354) gel depicts splicing of image in order to remove irrelevant lanes (B) Analytical SEC chromatograms of POT1-TPP1-TIN2(1–354) (green), TRF2-RAP1 (red), and reconstituted shelterin (POT1-TPP1-TIN2(1–354) + (2×)TRF2-RAP1, yellow). (C) Direct telomerase assay to assess the biological activity of the purified recombinant POT1-TPP1-TIN2(1–354) complex on the enzymatic activity of the human telomerase. The double-stranded telomeric ligand (telo666) was pre-incubated alone (−) or with increasing quantities (5 to 100 nM, 201/7-fold serial dilution) of fully (POT1-TPP1-TIN2(1–354)-TRF2-RAP1) or partially (POT1-TPP1-TIN2(1–354) or TRF2-RAP1) reconstituted shelterin sub-complexes prior to proceeding with telomerase-catalysed primer extension. The reaction products were analyzed by denaturing PAGE and phosphorimaged. Telomeric repeats are indicated with black diamonds. The position of the 1-, 10-, 20- and 30-fold repeats is depicted as twin diamonds. Results show that when present in the reaction, the purified recombinant POT1-TPP1-TIN2(1–354) complex alone or when associated with TRF2-RAP1 increases stoichiometrically the telomerase processivity. As a control, TRF2-RAP1 alone does not alter substantially the telomerase processivity. (TIF) [file pone.0264073.s002.tif]

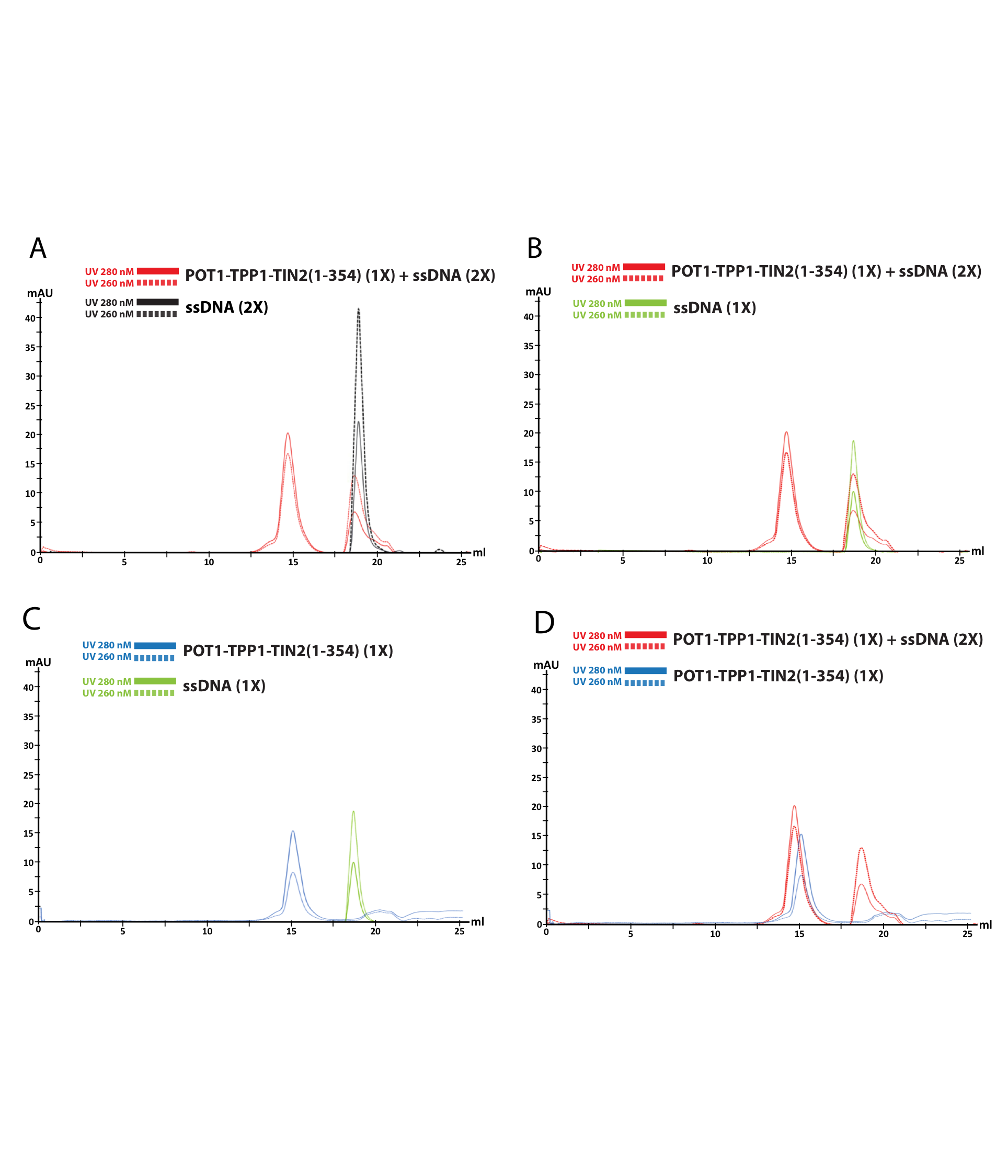

Supplement: S3 Fig — Straight lines represent the 280 nm absorbance and dotted lines represent the 260 nm absorbance. The blue traces represent the elution profile of an injection of 600 pmol (1×) POT1-TPP1-TIN2(1–354). The red traces represent the elution profile of an injection of 600 pmol (1×) POT1-TPP1-TIN2(1–354) incubated with 1200 pmol (2×) sstelo64 ssDNA ligand. The green traces represent the elution profile of an injection of 600 pmol (1×) sstelo64 ssDNA ligand. The black traces represent the elution profile of an injection of 1200 pmol (2×) sstelo64 ssDNA ligand. Comparison of the elution profiles are depicted as follows: (A) protein(1×)+ssDNA(2×) vs ssDNA(2×). (B) protein(1×)+ssDNA(2×) vs ssDNA(1×). (C) protein(1×) vs ssDNA(1×). (D) protein(1×)+ssDNA(2×) vs protein(1×). Comparison of the various elution profiles show that the POT1-TPP1-TIN2(1–354) complex binds approximately an equimolar quantity of its ssDNA ligand indicating a ~1:1 binding stoichiometry. (TIF) [file pone.0264073.s003.tif]

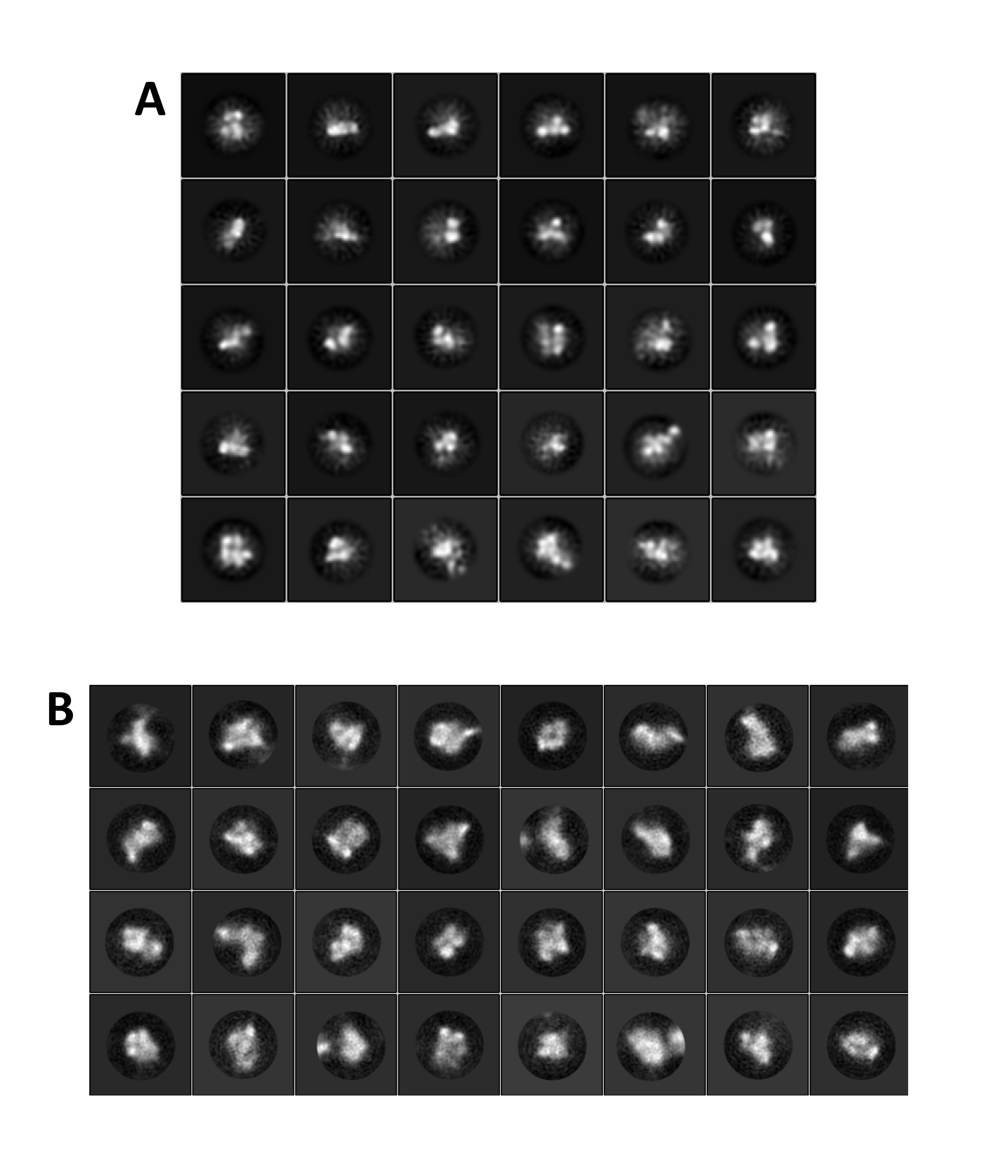

Supplement: S4 Fig — (A) Top 30 2D classes from cryo-EM of the fully assembled shelterin complex shows classes that are heterogenous and do not reconstruct into a reliable 3D map. (B) Top 32 2D classes from negative stain EM of the fully assembled shelterin complex shows similar representation of classes as in cryo-EM, which also suggests there is high conformational heterogeneity in the sample. (TIFF) [file pone.0264073.s004.tiff]

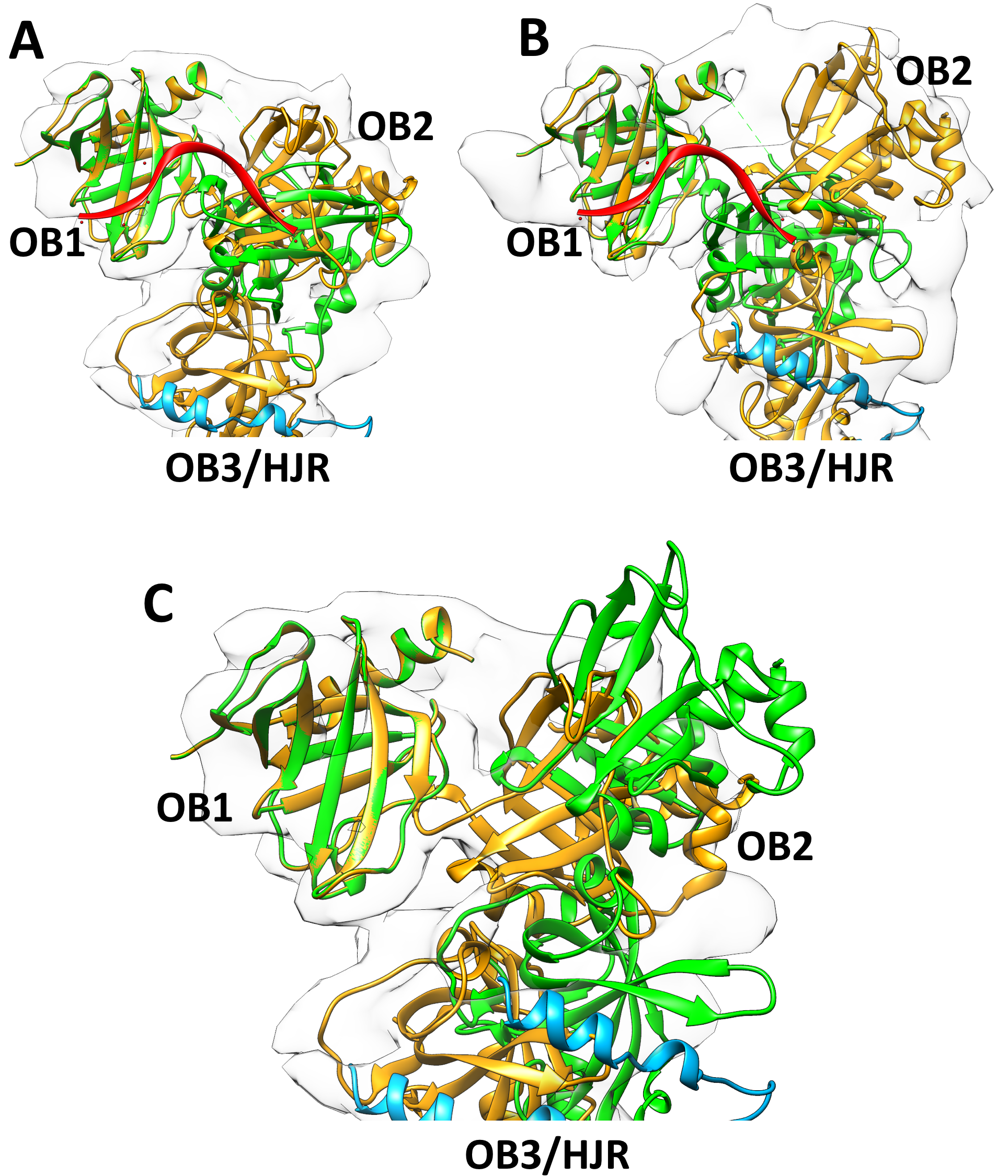

Supplement: S5 Fig — (A) Closed conformation of TPP1(blue)-bound POT1(yellow) structure superimposed to the X-ray structure (PDB ID: 1XJV) of the DNA(red)-bound POT1(green) OB1 and OB2 domains by aligning on the OB1 domain. The comparison between the two structures shows that the OB2 domains do not properly superimpose and that the OB2 domain in the DNA-bound crystal structure is sterically clashing with the OB3/HJR domain suggesting an alternative conformation likely forms in full-length POT1. (B) Open conformation of TPP1(blue)-bound POT1(yellow) structure superimposed to the X-ray structure (PDB ID: 1XJV) of the DNA(red)-bound POT1(green) OB1 and OB2 domains by aligning on the OB1 domain. The comparison between the two structures shows that the OB1 and OB2 domains are spaced apart in the open conformation resulting in a significant conformational difference between the two structures. (C) Superimposition on the OB1 domain of the closed (yellow) and open (blue) conformations of full-length POT1 complexed to the PBD of TPP1 observed through cryo-EM shows a significant difference in the position of the OB2 and OB3/HJR domains relative to the OB1 domain. (TIF) [file pone.0264073.s005.tif]
