# Supplementary material for: Insights into POT1 structural dynamics revealed by cryo-EM
Source: PLoS One. 2022 Feb 17;17(2):e0264073. doi: 10.1371/journal.pone.0264073 (PMC8853558; doi:10.1371/journal.pone.0264073)
Supplement: S6 Fig — The various 32P-labelled telomeric DNAs were incubated alone (−) or with increasing quantities (10 pM to 100 nM) of POT1-TPP1-TIN2(1–354) protein complex. The binding reaction products were separated by native PAGE. The radio-labelled telomeric DNA was detected by phosphorimaging. The filled and open arrows on the left denote the positions of the retarded protein-DNA complex and the free DNA, respectively. For each telomeric DNA construct investigated, a representative EMSA image is shown (upper panel) and quantification data of the unbound DNA fraction is depicted (lower panel) for all the independent experiments. Kd values were determined by non-linear regression and are shown in Fig 6B and 6C. The sequence of the single-stranded fragment of each ligand is depicted on the top of the panel and the complete sequence is available in S1B Fig. Investigated telomeric ligands are: (A) telo00, (B) telo60, (C) telo61, (D) telo62, (E) telo63, (F) telo64, (G) telo65, (H) telo66, (I) telo6^4, (J) telo6~4, (K) telo6¢4, (L) telo6^6, (M) telo6¢6, (N) telo6^, (O) telo6¢, (P) telo^4 and (Q) telo¢4. (PDF) [file pone.0264073.s006.pdf]

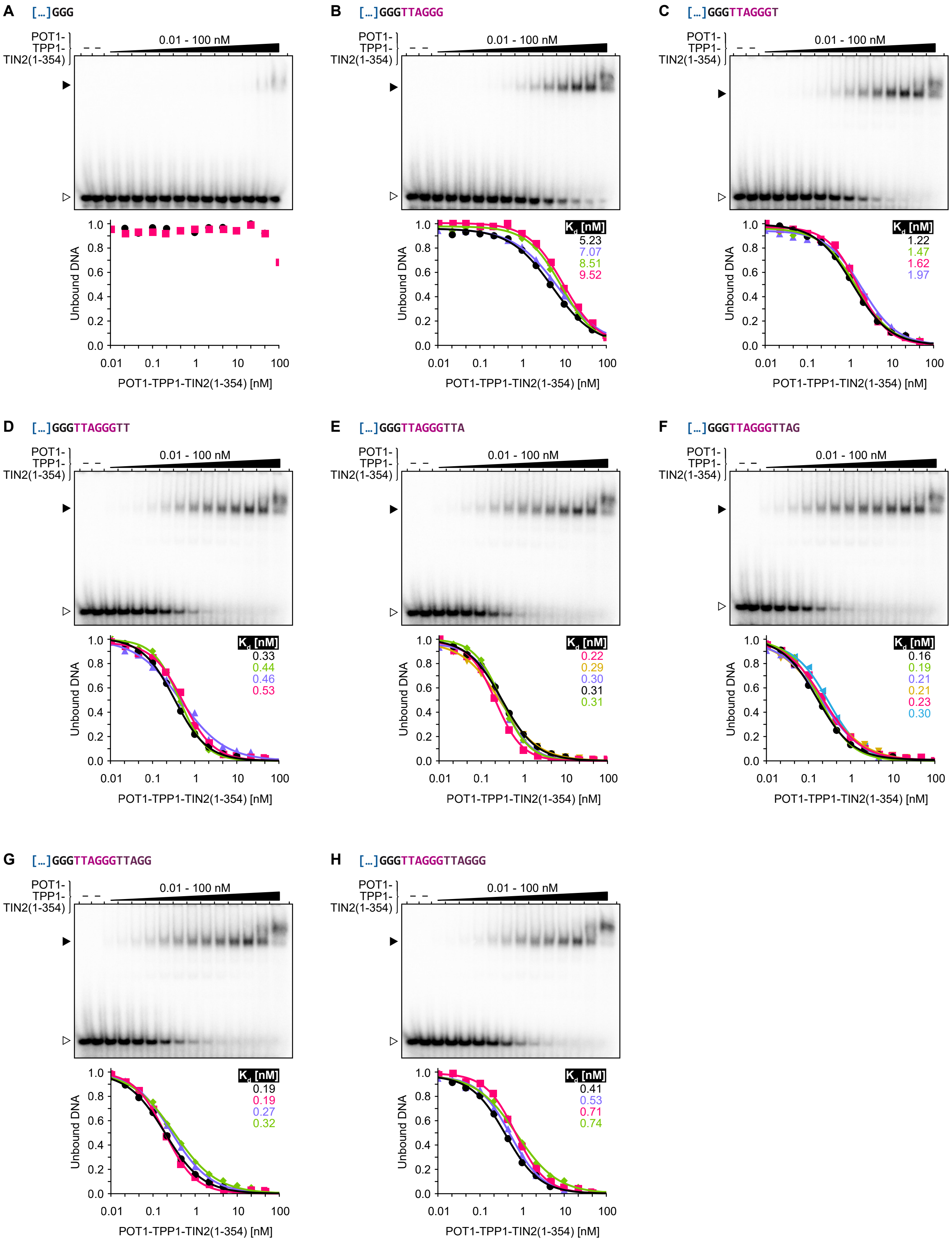
A [...]GGG
POT1-
TPP1-
TIN2(1-354)
0.01 - 100 nM
Unbound DNA
POT1-TPP1-TIN2(1-354) [nM]
B [...]GGGTTAGGG
Kd [nM]
5.23
7.07
8.51
9.52
C [...]GGGTTAGGGT
Kd [nM]
1.22
1.47
1.62
1.97
D [...]GGGTTAGGGTT
Kd [nM]
0.33
0.44
0.46
0.53
E [...]GGGTTAGGGTTA
Kd [nM]
0.22
0.29
0.30
0.31
0.31
F [...]GGGTTAGGGTTAG
Kd [nM]
0.16
0.19
0.21
0.21
0.23
0.30
G [...]GGGTTAGGGTTAGG
Kd [nM]
0.19
0.19
0.27
0.32
H [...]GGGTTAGGGTTAGGG
Kd [nM]
0.41
0.53
0.71
0.74

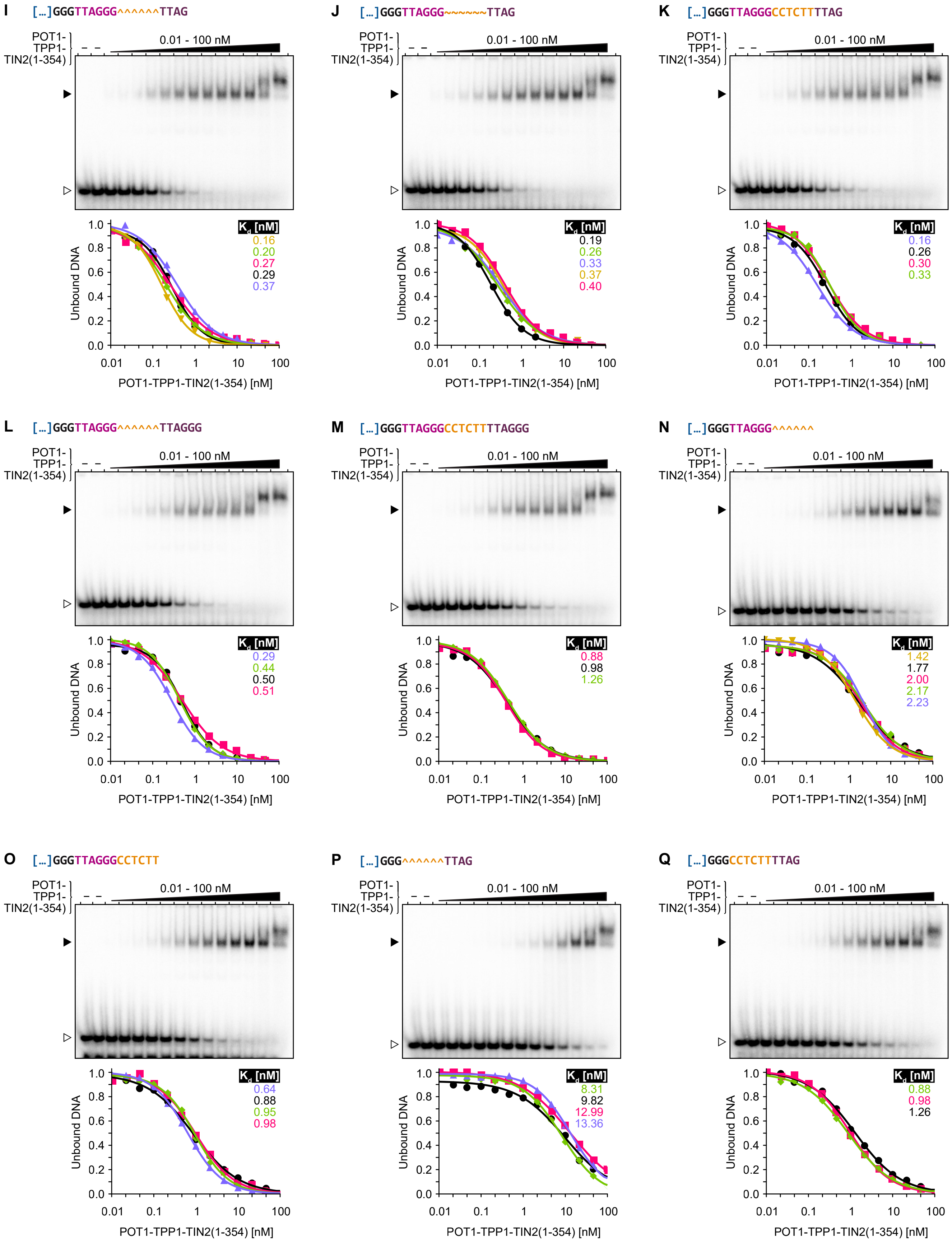
I [...]GGGTTAGGG^^^^^^TTAG
J [...]GGGTTAGGG~~~~~~TTAG
K [...]GGGTTAGGGCCTCTTTTAG
L [...]GGGTTAGGG^^^^^^TTAGGG
M [...]GGGTTAGGGCCTCTTTTAGGG
N [...]GGGTTAGGG^^^^^^
O [...]GGGTTAGGGCCTCTT
P [...]GGG^^^^^^TTAG
Q [...]GGGCCTCTTTTAG
POT1-
TPP1-
TIN2(1-354)
0.01 - 100 nM
Unbound DNA
POT1-TPP1-TIN2(1-354) [nM]
K$_d$ [nM]
I: 0.16 0.20 0.27 0.29 0.37
J: 0.19 0.26 0.33 0.37 0.40
K: 0.16 0.26 0.30 0.33
L: 0.29 0.44 0.50 0.51
M: 0.88 0.98 1.26
N: 1.42 1.77 2.00 2.17 2.23
O: 0.64 0.88 0.95 0.98
P: 8.31 9.82 12.99 13.36
Q: 0.88 0.98 1.26
