# Supplementary material for: Insights into POT1 structural dynamics revealed by cryo-EM
Source: PLoS One. 2022 Feb 17;17(2):e0264073. doi: 10.1371/journal.pone.0264073 (PMC8853558; doi:10.1371/journal.pone.0264073)
Supplement: S1 Fig — (A) Schematic representation of the prototypical shelterin ligand used in this work. Nearly all DNA ligands comprise both single-stranded and double-stranded telomeric regions with a 5′ 5-nt-long non-telomeric double-stranded extremity to ensure correct strand hybridisation register with the antisense DNA oligo ctelo. A 15-bp-long telomeric sequence enables the binding of TRF2 onto the 5′ side of the G-rich strand while a 10-to-12-nt-long single-stranded 3′ telomeric overhang constitutes the binding site for POT1. TRF2 and POT1 binding sequences are spaced by a 3-nt-long single-stranded telomeric sequence to limit steric hindrance between POT1 and TRF2. (B) Inventory and sequences of the DNA ligands used in this work. Double-stranded regions are underlined. The telo666 oligo used for the telomerase activity assay carries a G-to-C mutation (depicted as a lower-case letter) to prevent POT1 binding the 3′-extremity of the G-rich strand and to compete with telomerase binding [55]. The various type of spacer sequences are located between the OB1 and the OB2 site of POT1 and are depicted in orange. Spacer sequences consist of six residues of either: a C3 propanediol spacer (^), an abasic tetrahydrofuran (1′,2′-dideoxyribose) spacer (~) or a non-telomeric hexad (CCTCTT) sequence. (PDF) [file pone.0264073.s001.pdf]

A

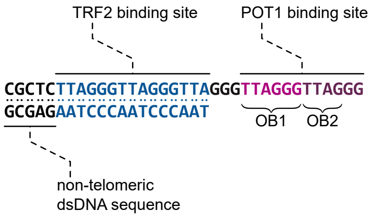

B

Name	Sequence (5′ → 3′)	Application	Used in (Fig.)
sstelo64	GGTTAGGGTTAG	SEC	S3
telo666	CGCTCTTAGGGTTAGGGTTAGGGTTAGGGTTAGcGTTAGGG	Telomerase assay	S2C
telo00	CGCTCTTAGGGTTAGGGTTAGGG	EMSA	S6A
telo60	CGCTCTTAGGGTTAGGGTTAGGGTTAGGG	EMSA	S6B
telo61	CGCTCTTAGGGTTAGGGTTAGGGTTAGGGT	EMSA	S6C
telo62	CGCTCTTAGGGTTAGGGTTAGGGTTAGGGTT	EMSA	S6D
telo63	CGCTCTTAGGGTTAGGGTTAGGGTTAGGGTTA	EMSA	S6E
telo64	CGCTCTTAGGGTTAGGGTTAGGGTTAGGGTTAG	EMSA, WEMSA	S6F, S7A
telo65	CGCTCTTAGGGTTAGGGTTAGGGTTAGGGTTAGG	EMSA	S6G
telo66	CGCTCTTAGGGTTAGGGTTAGGGTTAGGGTTAGGG	EMSA	S6H
telo6^4	CGCTCTTAGGGTTAGGGTTAGGGTTAGGG^^^^^^TTAG	EMSA	S6I
telo6~4	CGCTCTTAGGGTTAGGGTTAGGGTTAGGG~~~~~~TTAG	EMSA	S6J
telo6¢4	CGCTCTTAGGGTTAGGGTTAGGGTTAGGGCCTCTTTTAG	EMSA	S6K
telo6^6	CGCTCTTAGGGTTAGGGTTAGGGTTAGGG^^^^^^TTAGGG	EMSA	S6L
telo6¢6	CGCTCTTAGGGTTAGGGTTAGGGTTAGGGCCTCTTTTAGGG	EMSA	S6M
telo6^	CGCTCTTAGGGTTAGGGTTAGGGTTAGGG^^^^^^	EMSA	S6N
telo6¢	CGCTCTTAGGGTTAGGGTTAGGGTTAGGGCCTCTT	EMSA	S6O
telo^4	CGCTCTTAGGGTTAGGGTTAGGG^^^^^^TTAG	EMSA	S6P
telo¢4	CGCTCTTAGGGTTAGGGTTAGGGCCTCTTTTAG	EMSA	S6Q
ctelo	TAACCCTAACCCTAAGAGCG	Complementary strand	S2C, S6, S7
rand	TGCTCTGACACGAAATCTGCT	Random carrier ssDNA	S6, S7
