# Supplementary material for: Insights into POT1 structural dynamics revealed by cryo-EM
Source: PLoS One. 2022 Feb 17;17(2):e0264073. doi: 10.1371/journal.pone.0264073 (PMC8853558; doi:10.1371/journal.pone.0264073)
Supplement: S7 Fig — (A) POT1-TPP1-TIN2(1–354) complex integrity analysis by WEMSA. 32P-labelled telo64 telomeric DNA ligand at a concentration of 20 nM (upper panel) or 10 nM (middle and lower panels) was incubated alone (−) or with increasing quantities (10 pM to 100 nM) of POT1-TPP1-TIN2(1–354) protein complex. The binding reaction products were separated by native PAGE and electroblotted onto a PVDF membrane. The radio-labelled telomeric DNA was detected by phosphorimaging (left panels). The simultaneous presence of TIN2(1–354), TPP1 and POT1 proteins with the retarded mobility DNA bands was verified by probing the membrane with either anti-TIN2 (upper-middle panel), anti-TPP1 (central panel) or anti-POT1 (lower-middle panel) antibodies. Coincidence analysis of the ECL and 32P signals was obtained by stacking the phosphorimaging and immunoblotting data using the membrane corners as alignment references (right panels). The filled and open arrows denote the positions of the retarded protein-DNA complex and the free DNA, respectively. Data are representative of three independent experiments. (B) Western-blot analysis of the binding specificity of the anti-TIN2, anti-TPP1 and anti-POT1 antibodies. Proteins from 10-μl aliquots of the binding reactions shown in panel (A) were separated by SDS-PAGE, transferred onto a PVDF membrane and sequentially probed with anti-TIN2, anti-TPP1 and anti-POT1 antibodies. Positions and sizes (kDa) of marker proteins are shown at the left. (PDF) [file pone.0264073.s007.pdf]

**A**

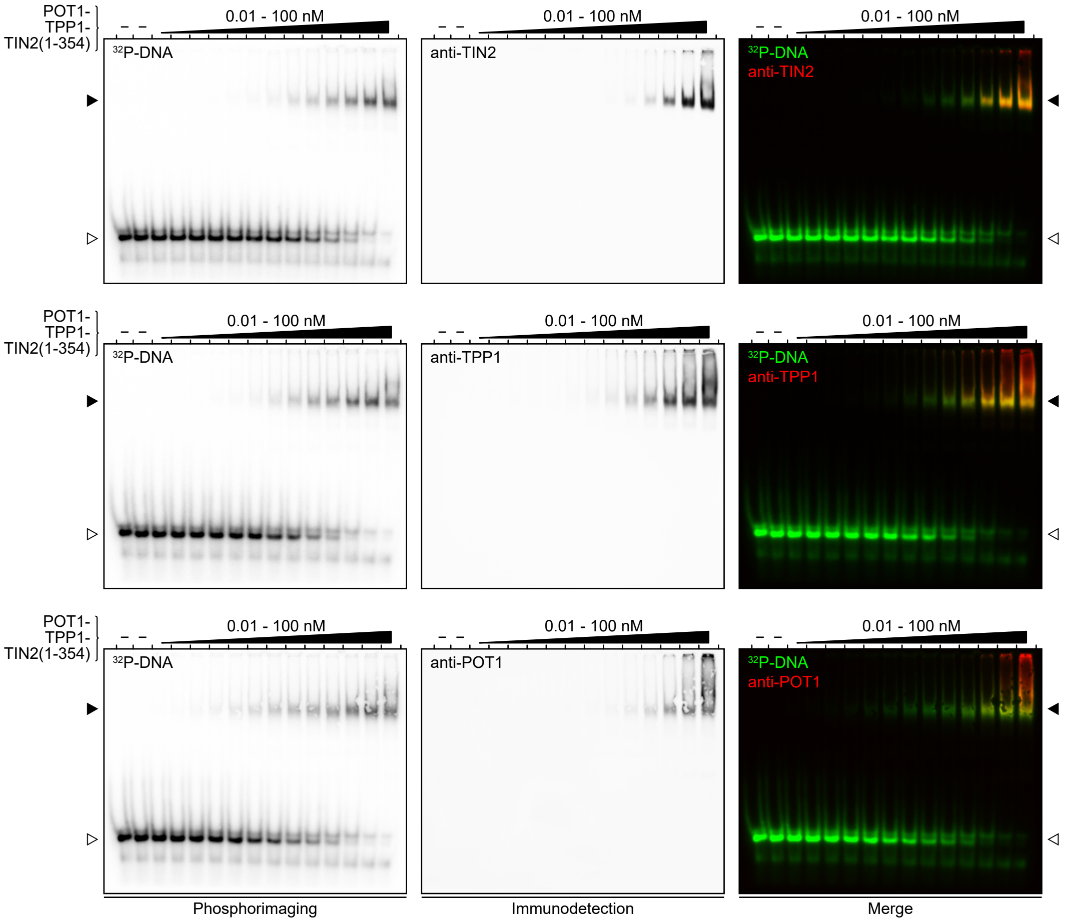
POT1-
TPP1-
TIN2(1-354)
0.01 - 100 nM
$^{32}$P-DNA
anti-TIN2
anti-TPP1
anti-POT1
Phosphorimaging
Immunodetection
Merge

**B**

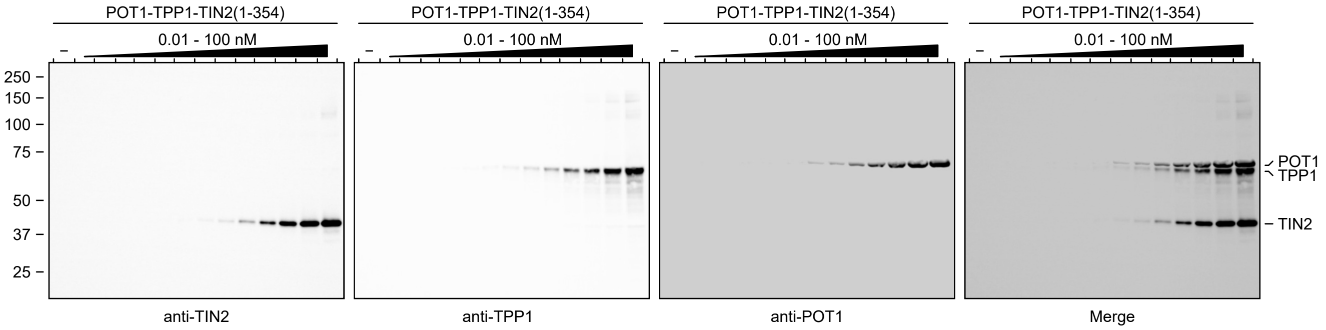
POT1-TPP1-TIN2(1-354)
0.01 - 100 nM
250
150
100
75
50
37
25
POT1
TPP1
TIN2
anti-TIN2
anti-TPP1
anti-POT1
Merge
